# Immune Signature Linked to COVID-19 Severity: A SARS-Score for Personalized Medicine

**DOI:** 10.3389/fimmu.2021.701273

**Published:** 2021-07-12

**Authors:** Jules Russick, Pierre-Emmanuel Foy, Nathalie Josseaume, Maxime Meylan, Nadine Ben Hamouda, Amos Kirilovsky, Carine El Sissy, Eric Tartour, David M. Smadja, Alexandre Karras, Jean-Sébastien Hulot, Marine Livrozet, Antoine Fayol, Jean-Benoit Arlet, Jean-Luc Diehl, Marie-Agnès Dragon-Durey, Franck Pagès, Isabelle Cremer

**Affiliations:** ^1^ Centre de Recherche des Cordeliers, Sorbonne Universite, Inserm, Universite de Paris, Team Inflammation, Complement and Cancer, Paris, France; ^2^ Hopital Europeen Georges Pompidou, AP-HP, Paris, Universite de Paris, Paris, France; ^3^ Centre de Recherche des Cordeliers, Sorbonne Universite, Inserm, Universite de Paris, Team Integrative Cancer Immunology F-75006, Paris, France; ^4^ Sorbonne Universite, Paris, France; ^5^ Department of Immunology, Hôpital Europeen Georges Pompidou, AP-HP, Paris, France; ^6^ Université de Paris, Innovative Therapies in Hemostasis, INSERM, Hematology Department and Biosurgical Research Lab, (Carpentier Foundation) Assistance Publique Hôpitaux de Paris, Centre-Université de Paris (APHP-CUP), Paris, France; ^7^ F-CRIN INNOVTE, Saint-Étienne, France; ^8^ Department of Nephrology, Hopital Europeen Georges Pompidou, AP-HP, Paris, France; ^9^ Department of Nephrology, Universite de Paris, Paris, France; ^10^ Université de Paris, INSERM, PARCC, Paris, France; ^11^ CIC1418 and DMU CARTE, AP-HP, Hôpital Européen Georges-Pompidou, Paris, France; ^12^ Department of Internal Medicine, Hopital Europeen Georges Pompidou, AP-HP, Paris, France; ^13^ Université de Paris, Innovative Therapies in Haemostasis, INSERM, Paris, France; ^14^ Intensive Care Unit and Biosurgical Research Lab (Carpentier Foundation), AH-HP, Georges Pompidou European Hospital, Paris, France

**Keywords:** COVID-19, immunologic profile, personalized medicine/personalized health care, score, therapeutic strategy

## Abstract

SARS-CoV-2 infection leads to a highly variable clinical evolution, ranging from asymptomatic to severe disease with acute respiratory distress syndrome, requiring intensive care units (ICU) admission. The optimal management of hospitalized patients has become a worldwide concern and identification of immune biomarkers predictive of the clinical outcome for hospitalized patients remains a major challenge. Immunophenotyping and transcriptomic analysis of hospitalized COVID-19 patients at admission allow identifying the two categories of patients. Inflammation, high neutrophil activation, dysfunctional monocytic response and a strongly impaired adaptive immune response was observed in patients who will experience the more severe form of the disease. This observation was validated in an independent cohort of patients. Using *in silico* analysis on drug signature database, we identify differential therapeutics that specifically correspond to each group of patients. From this signature, we propose a score—the SARS-Score—composed of easily quantifiable biomarkers, to classify hospitalized patients upon arrival to adapt treatment according to their immune profile.

## Introduction

Coronavirus disease 2019 (COVID-19), due to severe acute respiratory syndrome coronavirus 2 (SARS-CoV-2) infection, has affected over 118 million people and is responsible for 2.6 million deaths since the beginning of the pandemic (WHO, March, 11 2021). The clinical evolution of patients with SARS-CoV-2 infection is highly variable between individuals, ranging from asymptomatic state for the majority of patients to severe symptoms. Approximately 10 to 20% patients require hospitalization and intensive care units (ICU) admission mainly for acute respiratory distress syndrome (ARDS) or multi-organ failure (1, 2). Some factors increase the risk of COVID-19 severity comprising old age, male gender and cardiovascular comorbidities—diabetes, obesity and hypertension (3).

In severe COVID-19 patients, profound dysregulated immune responses have been described, characterized by strong systemic inflammation leading to acute injury of several organs including the lungs, the kidney and the heart (4–9). Severe or fatal COVID-19 is indeed associated with elevated innate pro-inflammatory immune cytokines in peripheral blood including interleukin (IL)-1, IL-6, IL-8, or C–X–C motif chemokine ligand 10 (CXCL-10) (7, 10) and alterations of both innate and adaptive immunity (11–13). Patients with COVID-19 have profoundly impaired induction of types I and III IFNs, that lead to untuned antiviral response and viral persistence (14). Dysfunctional type I IFN immunity have been attributed to either inherited intrinsic genetic defects in double-stranded RNA sensor TLR3 and interferon regulatory factor 7 (IRF7) (15), or to the production of neutralizing auto-Abs against type I IFNs (16) in respectively, 3.5 and 10.2% of life-threatening COVID-19 patients. Severe dysfunctions in neutrophils and monocyte populations (17–20), lymphopenia and uncoordinated responses of the three arms of SARS-CoV-2 specific adaptive immunity (CD4^+^, CD8^+^ T cell responses and B cell antibody production) were reported in patients with acute COVID-19, particularly in patients >65 years old (21, 22).

While COVID-19 severity is associated with immune disorders, there is a lack of robust biomarkers that identify at admission groups of hospitalized patients who will experience poor clinical outcome. In addition, several immunomodulators (dexamethasone and anti-IL-6R) are currently proposed to patients, with controversies with respect to their efficacy and there is no consensus about the use of these immunomodulatory drugs (23–27). The use of dexamethasone for up to 10 days was however shown to reduce 28-day mortality compared to usual care in patients needing oxygen or receiving invasive mechanical ventilation at randomization (28).

The aim of the present study was to segregate and characterize hospitalized COVID-19 patients based on their immune profile. We therefore performed an extensive analysis of immune parameters on a cohort of hospitalized COVID-19 patients, integrating flow cytometry, transcriptomic data, and multiple clinical variables reflecting organ damages. We identified two groups of patients based on immune gene expression, that segregate with disease severity. We propose a combined association of easily quantifiable biomarkers, called “SARS-Score” that allow an accurate classification of the patients. Finally, after validation of the signature on public data, we used *in silico* tools to propose a personalized medicine in COVID-19, that could specifically correspond to each group of patients.

## Materials and Methods

### Patient’s Cohort

A prospective observational cohort study of 36 adult patients (≥18 years old) with available samples admitted in the Georges Pompidou European Hospital (Paris, France) since March 2020 was analyzed in this study. All patients were diagnosed with COVID-19, i.e. positive for SARS-CoV-2 nucleic acid on real-time reverse transcription-polymerase chain reaction (RT-PCR) assays of nasopharyngeal swab specimens, in accordance with the World Health Organization (WHO) COVID-19 technical guidance (https://apps,who,int/iris/handle/10665/330854). On admission, all patients required oxygen and 27 were admitted in the ICU with the need of invasive mechanical ventilation. This study was approved by the medical ethic committee (CERAPHP·5 approval number 00011928). Patients included in the present study, or their relatives, were informed that their medical data could be used for research purposes in accordance with the General Data Protection Regulation (EU 210 2016/679). Clinical and biological variables were obtained at admission, reflecting lung, liver, renal and cardiac function and hemostasis (**Supplementary Table 6**).

For each patient, the day after admission, blood was collected in PAXgene tubes (Promega, Madison, WI, USA) for further RNA extraction, or in citrate tubes for flow cytometry analyses. Plasma was frozen for assay of cytokine levels, and peripheral blood mononuclear cells (PBMC) were isolated and frozen for further flow cytometry analysis. Samples from non-infected control subjects were purchased from the French Blood Establishment.

### Flow Cytometry

Thawed cells were stained with live/dead for viability (Thermofisher, Waltham, MA, USA), monoclonal antibodies (mAbs) directed against CD3, CD209 (Beckman Coulter, Brea, CA, USA), CD14, CD16, CD19, CD56, CD86, CD299 (BD Biosciences), CD8, CD40, HLA-DR (BioLegend, San Diego, CA, USA) and CD163 (Miltenyi Bergisch Gladbach, Germany), for 30’ at 4°C in PBS and 10% FCS medium. Staining was acquired on Fortessa X20 (BD Biosciences, San Jose, CA, USA) and analyzed using FlowJo software.

The unsupervised analysis was done using Excyted pipeline (https://github,com/maximemeylan/Excyted). Intensity values of events gated from live cells were normalized using the Logicle transformation. Unsupervised clustering and Uniform Manifold Approximation and Projection (UMAP) were computed with 10,000 events for each sample using k = 30.

### Luminex

Plasma cytokines were measured by Luminex technology (Bio-Plex, Bio-Rad, 27-Plex Assays panel, Marnes-la-Coquette, France) according to the manufacturer’s instructions.

### RNA Extraction

Total RNA was purified from frozen PBMCs of COVID-19 patients using the Maxwell 16 LEV simplyRNA Cells Kit (Promega, Madison, WI, USA), according to the manufacturer’s instructions. Cell pellets were dispersed in the chilled 1-Thioglycerol/Homogenization Solution. Total RNA was eluted in a low volume of 50 μl, RNA quality and quantity were estimated on a NanoChip (Total Eukaryote RNA Assay Nano II Kit, Qiagen, Düsseldorf, Germany) by capillary electrophoresis (BioAnalyzer, Agilent Technologies, Santa Clara, CA, USA). Samples with a RIN ≥8 were considered suitable for Reverse Transcription and Real-Time PCR experiments.

### Reverse Transcription

Reverse-transcriptions were carried out on the entire RNA sample in a 20 μl total reaction volume with the High-Capacity cDNA Reverse Transcription Kit with RNAse inhibitor (PN 4368814, Applied Biosystems, Waltham, MA, USA) according to the manufacturer’s instructions. Concentration of cDNA was estimated with a Qubit 3.0 Fluorometer (Q33216, Thermofisher).

### Semi-Quantitative Real-Time Polymerase Chain Reaction

Real-time PCR were performed with 40 ng of cDNA using the 2× TaqMan Universal Master Mix (Applied Biosystems) and 20× Taqman^®^ Gene Expression Assay for the detection of human targets TLR3, TLR7, RIG-I, and MDA-5. Reactions took place in a Hard-Shell 384-well PCR plate (Biorad, Hercules, CA, USA) in a 10 μl total reaction volume. Detection and semi-quantification of gene expression were performed on a CFX384 Touch™ Real-Time PCR Detection Instrument (Biorad). Technical triplicates were carried out. Semi-quantitative real-time PCR results were analyzed with the dedicated CFX Manager software (Biorad). Data standardization was carried out using the Ct obtained for the Human GAPDH housekeeping gene. The ΔCt were used to evaluate the differential expressions by estimation of the Fold Change.

### nCounter^®^ Gene Expression

The kit used for the Gene Expression analysis was the nCounter^®^Cancer Immune™ Panel (NanoString Technologies, Seattle, WA, USA). A master mix was created by adding 70 μl of hybridization buffer to cancer immune reporter code set tube. Hybridization step was performed in the nCounter 12-well Notched Strip Tubes. The following components were added respectively: 8 μl of MasterMix, 100 ng of RNA in a volume of 5 μl, 2 μl of capture probe set. The tubes were immediately incubated 21 h in a pre-heated 65°C thermal cycler. Once removed from the thermal cycler, hybridization reactions were immediately processed with the nCounter Prep-station for purification step and immobilization in a cartridge. Finally, data collection was carried out in the nCounter^®^ Digital Analyzer (FOV555).

### Nanostring nCounter Data Analysis

Quality control of nCounter data was performed with the nSolver software developed by NanoString Technologies. Samples with insufficient detection limit were excluded from the analysis. A hierarchical clustering was performed on the normalized nCounter data using Euclidean distance and the Ward method. Normalization was performed with the package NanoStringNorm (29). The nCounter data embed positives (6), negatives (8) and housekeeping genes (30) required to normalize data. The package gplots was used to generate a heatmap showing the hierarchical clustering and the relative genes expression. Principal component analysis on normalized nCounter data was performed with the package factoextra.

Differential gene expression analysis based on NanoString nCouter data was performed using the Nanostringdiff package version 1.20.0 (available from Bioconductor) (31). The normalization procedure and the identification of differentially expressed genes between groups were done using methods provided by the package. The adjusted p-values (q-values) were calculated using the Benjamini and Hochberg procedure (false discovery rate). Genes with q-value <0.05 and a fold change greater than 2 (log2 fold change >1) or less than −2 (log2 Fold change <−1) have been identified as overexpressed genes or underexpressed genes respectively. Volcano plot showing the results was generated with the package EnhancedVolcano.

### Gene Enrichment Analysis

Enrichment analysis was performed using the package EnrichR downloaded from CRAN (32, 33). For tissue and cell signatures we used The Human Genome Atlas. For biological process and pathway signatures we used KEGG 2019 and Gene Ontology Biological Process 2018. For drug signatures we used DsigDB and signatures from the GEO Drug perturbation. Signatures were ordered according to the q-values and only the more significant signatures were considered relevant (q-values <0.05).

### Public RNAseq Data (GSE157103)

In order to confirm our results on a larger cohort, we downloaded the TPM normalized RNA seq data from the GSE157103 dataset (34). The data include transcriptomic data from 100 COVID-19 patients (whole blood) and corresponding clinical annotations.

### Statistical Analyses

The statistical analysis was performed using R 4.0.2 and appropriate packages available from CRAN or Bioconductor. For quantitative variables, we used the Mann–Whitney–Wilcoxon test to compare the distribution between two groups. For correlation analysis, we used the Spearman method. Correlation coefficient and p-values was calculated with the package hmisc and correlogram was generated with the package corrplot.

## Results

### COVID-19 Patient Clinical Characteristics, Immunophenotyping and Gene Expression Signature

We analyzed a prospective cohort of 36 hospitalized COVID-19 patients (mean age of 62 years) with a clinical course ranging from moderate to severe disease, with nine patients hospitalized in the general ward and 27 needing ICU admission (**Supplementary Table 1**). We extensively collected clinical and routine laboratory tests on admission reflecting lung, liver, renal, cardiovascular functions and hemostasis.

We first compared the immune profiles of COVID-19 patients to those of 10 healthy donors (controls) by flow cytometry analysis of PBMCs (gating strategy shown in **Supplementary Figure 1**). We found lymphopenia with a decrease of both T and B cells numbers in COVID-19 patients, as previously reported (21), while no difference in NK and NKT cells **(** **Supplementary Figure 2A** **)**. Monocytes, are not different in numbers, but display high expression of CD16, CD40, CD163 and a low expression of HLA-DR compared to healthy donors meaning either an over-activated phenotype or a compensatory anti-inflammatory response as CD86 is not upregulated in COVID patients **(** **Supplementary Figures 2B, C** **)**. The expression level of the viral sensors TLR3, TLR7, DDX58 coding for RIG-I and interferon induced with helicase C domain 1 (IFIH1) coding for MDA-5 were highly variable among patients **(** **Supplementary Figure 2D** **).** TLR7 was the sole sensor significantly overexpressed in COVID-19 patients as compared to controls.

The most important difference being observed for monocytes, we performed an unsupervised analysis of the flow cytometry data on CD14^+^ cells. We identified 19 clusters (**Supplementary Figure 3A**) of which 16 are significantly differentially represented between the control group and COVID-19 patients (eight clusters more abundant and eight less abundant), and showing different expression profiles of monocytes markers as shown in the heatmap representation (**Supplementary Figure 3B**). For each COVID-19 patient, we then correlated the percentages of each differentially represented cluster with the levels of cytokines and chemokines that were evaluated by luminex assay, markers of blood vessels and of organs dysfunctions and viral sensors expression. We found that over-represented clusters are correlated with inflammatory cytokines and chemokines (IL-6, IL-18, sIL-6Ra, sTNFR1, CXCL10, CCL2 and eotaxin) and markers of vessel inflammation, whereas a negative correlation was found between the under-expressed clusters and these inflammatory molecules (**Supplementary Figure 3C**).

Altogether, these data showed a distinct immune profile of COVID-19 patients as compared to healthy donors with a strong heterogeneity among hospitalized COVID-19 patients, both in terms of immune populations, activation status and viral sensors expression.

### Immune Signature Identifies Two Groups of COVID-19 Patients

**Figure 1:**
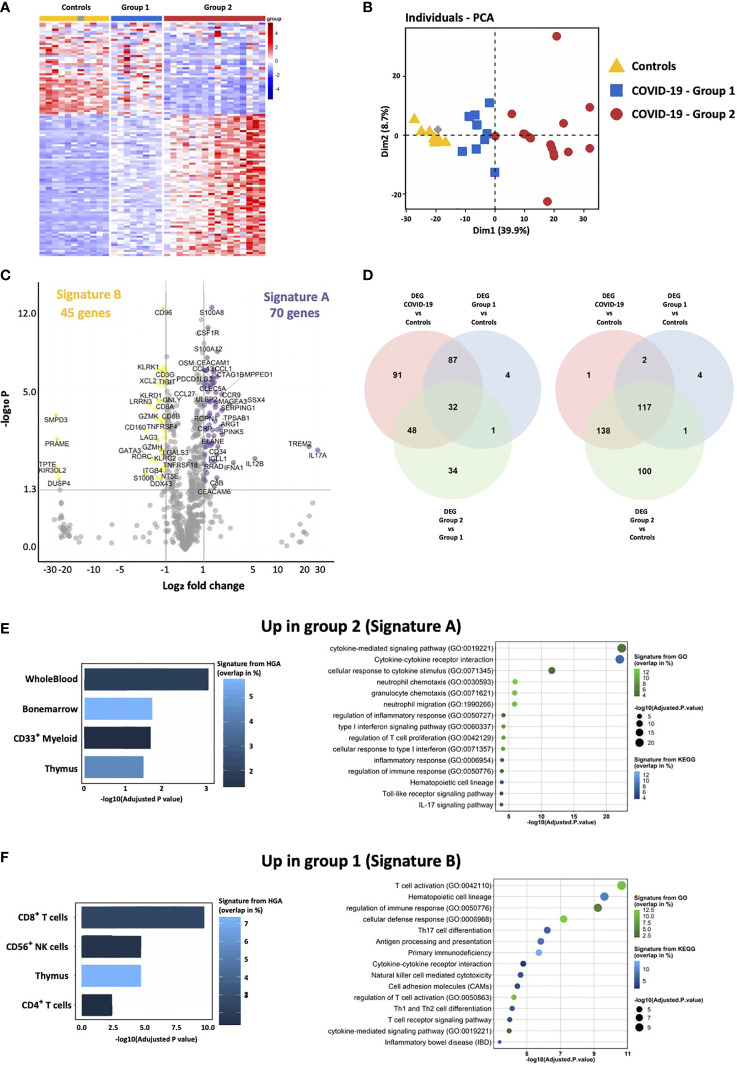
Transcriptomic immune gene signature identifies two groups of COVID-19 patients. **(A)** Heatmap representation showing relative expression of immune genes by COVID-19 and controls reveals the presence of two groups of patients: group 1 (blue) and group 2 (red). **(B)** Principal component analysis of COVID-19 and controls shows that group 1 is intermediate between controls and group 2 COVID-19 patients. **(C)** Volcano Plot showing differentially expressed genes between group 2 and group 1. X axis displays fold changes between the two groups and Y axis the −log_10_ (p value). Differentially overexpressed genes (Signature A—highlighted in purple) and under-expressed genes (Signature B—highlighted in yellow) by group 2 as compared to group 1 patients were characterized by fold changes superior/inferior to 2 and with a significant p value (<0.05). **(D)** Venn diagrams showing common differentially expressed genes between group 1, group 2 and controls. **(E, F)** Enrichment analysis of genes of the signatures A and B (respectively up-regulated and down-regulated in group 2) using EnrichR and three different datasets. The histogram shows the first five more significant enriched signatures from The Human Genome Atlas (HGA). corresponding to tissue and cell signatures. The bubble plot shows the first 15 more significant enriched signatures from the Kyoto Encyclopedia of Genes and Genome (KEGG) and Gene Ontology Biological process (GO), corresponding to biological pathway signatures. Signatures are ordered according to adjusted p-values, the color graduation shows the percentage of genes overlapping between the datasets signature and our own signature.

Hierarchical clustering of transcriptomic analysis of 730 immune related genes from PBMC of COVID-19 patients with available samples (n = 25) at admission and of 10 healthy donors, revealed a complete segregation between controls and patients, except for one patient who developed a moderate disease (WHO score = 4) with a short non-ICU hospitalization (**Figure 1A**). Overall, 35% of the immune related genes explored were differentially expressed between patients and controls, with 178 genes overexpressed and 80 genes under-expressed (**Supplementary Tables 2** and **3**).

Strikingly, hospitalized COVID-19 patients segregated into two groups 1 and 2 **(** **Figure 1A** **)**. Principal component analysis of transcriptomic features of whole blood at the onset of hospitalization revealed that group 1 (blue) was closer to healthy controls than group 2 (red) (**Figure 1B**). Delay between the onset of symptoms and sample collection (performed day 2 of hospitalization) was identical in the two groups (9.5 and 9.9 days for groups 1 and 2, respectively, P-value = 0.3672), indicating that transcriptomic differences were not due to temporal discrepancies. Seventy genes (i.e. signature A) and 45 genes (i.e. signature B) were significantly over-expressed and under-expressed, respectively, in group 2, as compared to group 1 patients (**Figure 1C** and **Supplementary Tables 4, 5**). Using the Venn-diagram representation, we identified that among the 39 genes differentially expressed between group 1, group 2 and controls, 34 belong to the group 2 and only four to the group 1, which confirms that group 2 is characterized by a specific gene expression signature. Moreover, we also showed that group 2 display the most important difference in number of genes differentially expressed between all COVID patients, or each group and controls **(** **Figure 1D** **)**. Genes up-regulated in group 2 belong to signatures of myeloid cells, neutrophil activity, inflammatory response, TLR and type I IFN signaling pathways, and inhibition of T cell proliferation (i.e. arginase 1, PDL1, PDL2 and CD276/B7-H3), (**Figure 1E** and **Supplementary Table 4**). At the opposite, genes involved in CD8 and NK cell function, T cell activation, T helper (Th) differentiation, co-stimulatory receptors (TNFRSF4 (OX40), ICOSLG (ICOS ligand), TNFRSF18 (GITR) and TNFRSF11A (RANK)), and antigen presentation, were down-regulated in group 2 (signature B; **Figure 1F** and **Supplementary Table 5**). These results are compatible with the coexistence of patients with a distinct immune profile: (i) adaptive immune response triggering (i.e. group 1), (ii) exacerbated myeloid and innate responses, with dysfunctional adaptive immune response (i.e. group 2).

### Clinical Outcome in Patient’s Groups According to Immune Signature

**Figure 2:**
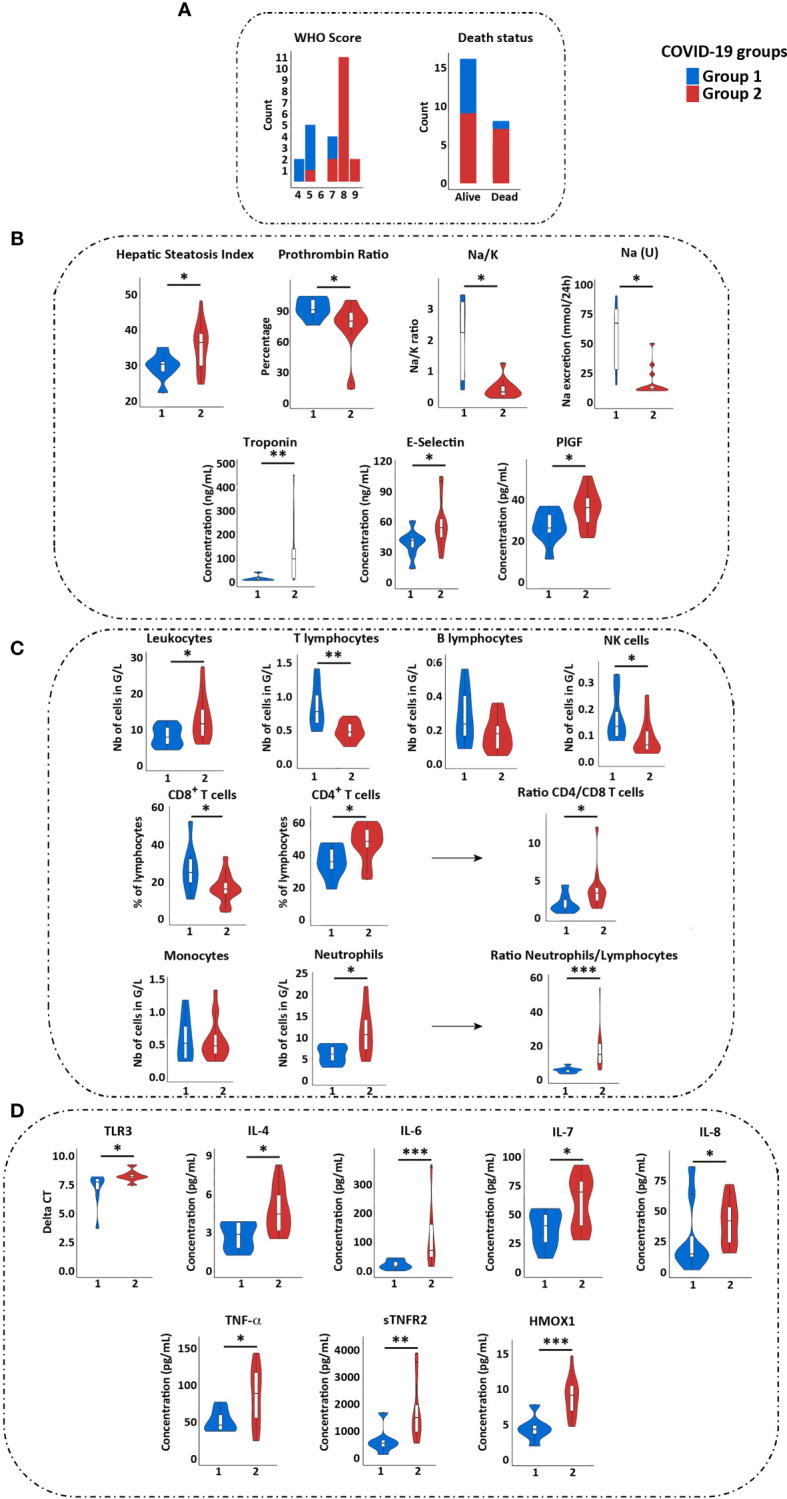
Immune and clinical characteristics of groups 1 and 2 COVID-19 patients. **(A)** Comparison of WHO score, of need for ICU hospitalization and of death status between group 1 (blue) and 2 (red) patients. **(B)** Comparison of clinical values reflecting liver (HSI and prothrombin ratio), renal [Na/K and Na (U)], cardiac function (troponin), and blood vessels (E-selectin and PIGF) between groups 1 and 2 patients. Only the values showing significant differences between the two groups are shown. **(C)** Comparison of immune cells quantification in groups 1 and 2 patients. **(D)** Comparison of TLR3 expression and cytokines quantification in two groups of patients (by Luminex assay). Statistical analyses were performed by Wilcoxon test using GraphPad software. *p < 0.05; **p < 0.01; ***p < 0.001. ICU, Intensive Care Unit; PlGF, Placental Growth Factor.

Patients of group 2 did not differ from group 1 for age and diabetes, but have a higher body mass index compared to group 1 (**Supplementary Table 6**). The severity of respiratory distress in each group was estimated by the WHO score. Patients without oxygen therapy, oxygen by mask, or nasal prongs had a 4–5 WHO score, whereas patients requiring oxygen by NIV or high flow, mechanical ventilation or extracorporeal membrane oxygenation (reflecting a severe lung damage) had >6 WHO score. Strikingly, almost all patients (15/16; 94%) from group 2 had severe respiratory distress (>6 WHO score) as compared to 25% (2/8) in group 1 (P = 0.0013) (**Figure 2A**). Differential biological variables between the two groups, reflecting other vital organs and tissues (i.e. liver, kidney, heart and blood vessels) (**Supplementary Table 6**) were investigated. Among them Hepatic Steatosis Index (HSI) and prothrombin ratio (% PR) reflecting liver damage, urinary Na^+^ and Na^+^/K^+^ reflecting kidney injury, troponin for cardiac damage, and E-selectin and placental growth factor (PlGF) reflecting the vessel status were significantly different in group 2 patients as compared to group 1 (**Figure 2B**). Overall, multi-organ failure, potentially exacerbated by endothelium damage and thrombosis was more pronounced in group 2 patients. Finally, 88% (7/8) of death belongs to group 2 **(** **Figure 2A** **)**, underlying distinct clinical outcome associated with differential immune patterns of patients’ groups.

We then compared the immune populations between both groups of COVID-19 patients. The group 2 was characterized by increased total leucocytes counts, with a profound lymphopenia, a decreased proportion of CD8^+^ T cells and increased proportion of CD4^+^ T cells, a decrease in NK cells and an increase of neutrophils (**Figure 2C**). In addition, group 2 patients presented with higher plasma level of proinflammatory cytokines (IL-6, IL-8, TNF-α, soluble TNF receptor 2 (sTNFR2)), of IL-4 and IL-7, and of anti-inflammatory heme oxygenase 1 (HMOX1) (**Figure 2D** and **Supplementary Table 7**). These data confirmed a higher inflammatory response, neutrophilia and reduced NK and T cell responses in group 2, whereas group 1 had a profile favoring an adaptive immune response. Of note, group 2 patients displayed a higher expression of TLR3 on PBMC (with no difference for TLR7, RIG-I and MDA-5) **(** **Figure 2D** **)**.

### Immune Gene Signature Identifies a Similar Group of Severe Patients in Public Cohort

In order to confirm our results, gene signatures A and B, on which we based the definition of patient’s groups with distinct prognosis, we investigated an independent public cohort of 100 hospitalized COVID-19 patients, with available RNAseq (GSE157103) and clinical data sets. All the genes of the signature were not detected in the dataset, so we first verified whether the expressed genes of each signature were co-expressed in this public cohort dataset and performed a gene enrichment analysis to show that we keep the same enrichment signature. (**Supplementary Figure 4A**). This analysis confirmed that genes of the signature A correspond to myeloid and neutrophil signatures, complement and coagulation and negative regulation of T cell responses, whereas genes of the signature B correspond mainly to positive regulation of immune responses (antigen presentation, T and NK cell activation) (**Supplementary Figure 4B**).

**Figure 3:**
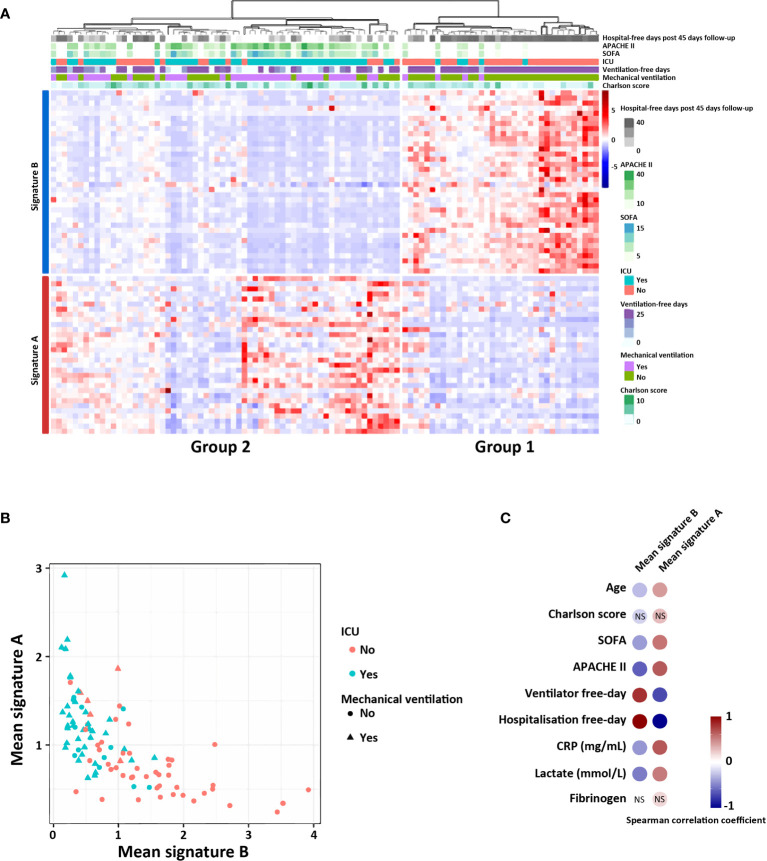
Signatures A and B identify two groups of patients in a public COVID-19 patient cohort (RNAseq data). **(A)** Heatmap showing relative expression of the gene signature and hierarchical clustering of COVID-19 patients with clinical and biological annotation. **(B)** Mean expression of the signatures A and B according to type of service hospitalization (ICU or not) and the need of mechanical ventilation (yes or not). **(C)** Significant correlation between the mean expression of—signatures A and B with the different clinical scores and biological values. Correlations were determined with the spearman correlation coefficient. NS, not significant.

Hierarchical clustering showed that gene signatures A and B segregated two groups of patients, as observed in our previous cohort (**Figure 3A**). Patients of group 2 compared to group 1 had higher comorbidity (Charlson score), longer duration of hospitalization, were more often hospitalized in ICU with higher ICU severity scores (APACHE II and SOFA), and required more often invasive mechanical ventilation (**Figure 3A**).

We finally calculated, for each patient, the correlation between the mean expression of signatures A and B and clinical and biological data related to severity of the disease. Interestingly, patients expressing signature B had no invasive mechanical ventilation and were not hospitalized in ICU, suggesting a milder form of COVID-19 infection **(** **Figure 3B** **)**. Biological markers of inflammation (CRP and fibrinogene) and of tissue injury (lactate), were also significantly correlated with the mean expression of signature A, which correspond to group 2 patients **(** **Figure 3C** **)**.

These data confirm that the immune signatures, determined the second day of hospitalization, were able to classify patients and predict distinct clinical evolution.

### Identification and Validation of a SARS-Score

**Figure 4:**
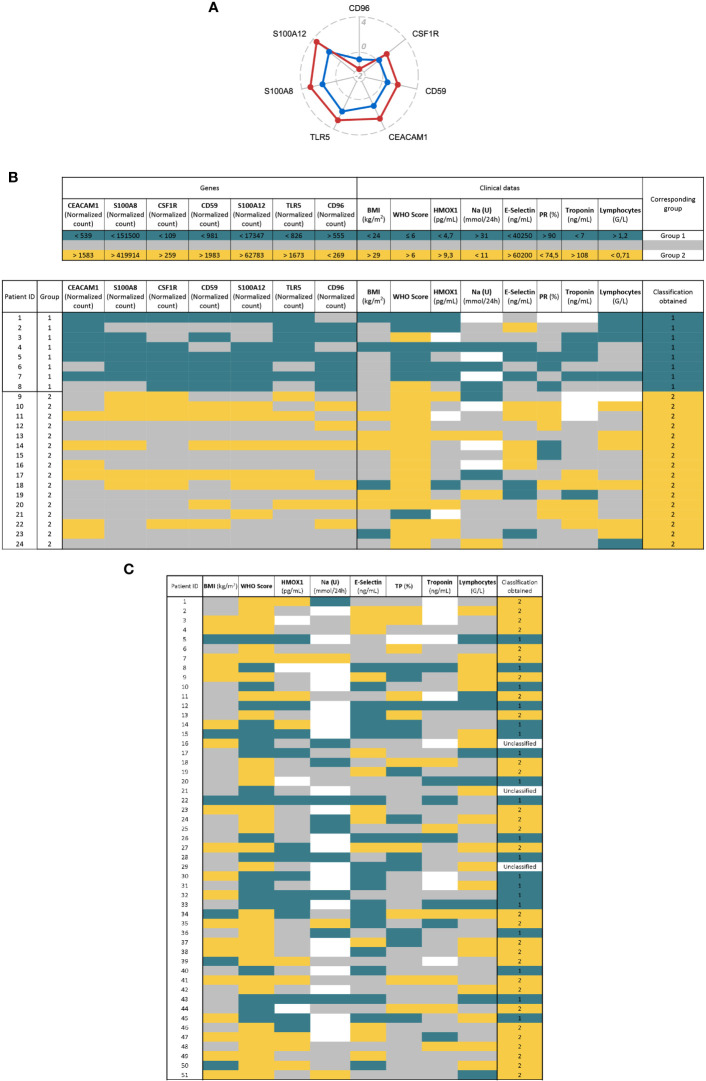
Definition of a SARS-Score for hospitalized patients and proposal of therapeutic agents. **(A)** Radar plot showing biomarkers from the signatures. We selected the genes with less than 10% of distribution overlap between the two groups of patients. The data shown are the log2 fold change of the mean of group 1 (blue) or group 2 (red) relative to controls. **(B)** The SARS-Score is composed of seven genetic (left) and eight clinical variables (right). For each variable, the upper part of the table displays the thresholds defined to classify patients in group 1 (blue) or 2 (yellow). These thresholds correspond to the first (25%) and last quartile (75%) of the total distribution, except for the WHO score which corresponds to the threshold between mild and severe disease (WHO Score = 6). The lower part of the table shows the application of the SARS-Score on our cohort. The blue and yellow cells correspond to values allowing a classification in group 1 or 2, respectively. Gray cells represent values that do not allow classification and white cells correspond to missing values. The final score (column “classification obtained”) is obtained by adding up the number of each colored cell. **(C)**. Application of the clinical part of the SARS-Score on 51 COVID-19 patients. The color code and the thresholds used are the same as in **(B)**. Patients having more blue or yellow cells are classified as “group 1” or “group 2”, respectively. Patient having the same number of blue and yellow cells are considered as “Unclassified”.

We then searched for a minimal combination of immune gene signature and of clinical biomarkers which would make it possible to determine which patients are most at risk to evolve into a poor clinical outcome. To obtain the minimal combination of genes, we selected the ones for which the expression was the most discriminant between groups 1 and 2 (**Figure 4A**). We thus identified seven genes (CEACAM1, S100A8, S100A12, CSF1R, TLR5, CD59 and CD96) with less than 10% of distribution overlap between the two groups of patients. In parallel, we determined among clinical data, a combination of eight clinical variables that are easily obtainable in routine. To create a stringent score, we defined for each clinical biomarker, a threshold corresponding to a classification in group 1 or 2 **(** **Figure 4B** **)**. These thresholds correspond to the extreme quartiles (first and last 25%) of the total distribution among the cohort. Combined together, these biomarkers constitute a powerful score, called SARS-Score, that could be useful to guide therapeutic management of severe patients.

We validated clinical part of this score on another cohort of 51 COVID-19 hospitalized patients (from HEGP hospital) and were able to classify 48 of them in group 1 or 2 (**Figure 4C**). We confirmed that group 2 patients of this cohort are characterized by a high WHO score (96.6% >6 for group 2, 10.5% >6 for group 1); p = 9.6e−09), ICU hospitalization (100% in ICU for group 2, 31.6% for group 1; p = 1.24e−07), high rate of death (41% for group 2, 21% for group 1; p = 0.12) (**Supplementary Figure 5**).

### Identification of Potential Therapeutic Targets

**Figure 5:**
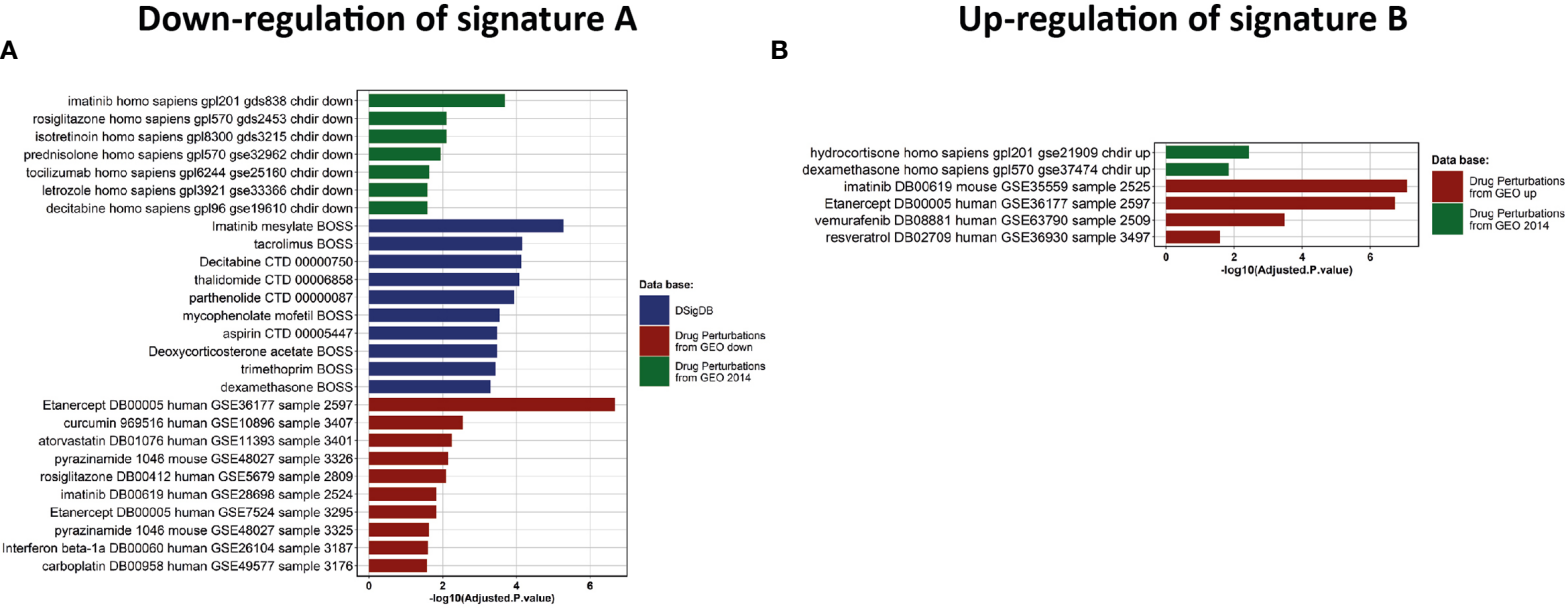
Drug discovery and clinical assays. **(A, B)** Enrichment analysis of drug signatures from DsigDB and GEO drug perturbations to down-regulate Signature A or up-regulate signature B, respectively.

We then investigated which drugs could be beneficial for each group of patients. To this end, we performed a drug-set enrichment analysis using EnrichR and drug signature databases (DsigDB (35) and GEO). Those databases include genes differentially expressed after drug treatment which induced a phenotype of interest by its action on known or unknown (off-target effects) targets, resulting in modification of gene expression. The objective of this approach is to identify drugs that would induce the downregulation of signature A (**Figure 5A**) and the upregulation of signature B (**Figure 5B**). Most of the drugs identified with this approach were immunomodulatory drugs. We found two inhibitors of cytokines: Tocilizumab (anti-IL-6 receptor) and Etanercept (anti-TNF-α receptor), glucocorticoids (dexamethasone. prednisolone and hydrocortisone), anti-inflammatory (aspirin, curcumin and parthenolide) and immunosuppressive (tacrolimus and mycophenolate) drugs. We also identify repurposed drugs with immunomodulatory properties like imatinib, thalidomide, isotretinoin, atorvastatin, vemurafenib and rosiglitazone. Interestingly, most of these drugs are being tested in clinical trials **(** **Table 1** **)** but further investigations are needed to confirm a potential benefit effect in COVID-19 patients. Nevertheless, these results highlight the importance of administering immunomodulatory therapies specifically to patients of group 2.

**Table 1:** Clinical trials using drugs that could down-regulate the signature A and up-regulate the signature B (last update: June 16, 2021).

Therapeutics	Drugs	Clinical trial (numbers)	Results		Population of COVID-19 patients	Ref
**Glucocorticoids**	Dexamethasone	Yes (58)	Clinical benefit	In combination with standard care, increase of ventilator-free days	All patients	NCT04327401
	Prednisolone	Yes (41)	Controversial	Early administration decrease death rate and ventilator dependence	Severe	NCT04323592
				Early short administration improves clinical outcomes	Moderate and severe	NCT04374071
				May prolong virus shedding	Severe	NCT04273321
				Early short administration don’t reduce mortality	All patients	NCT04343729
	Hydrocortisone	Yes (10)	No result published			
**Inhibitors of cytokines**	Etanercept	No	No clinical trial			
	Tocilizumab	Yes (57)	Controversial	No benefit on disease progression	All patients	NCT04346355
				Don’t improve clinical outcomes at 15 days, and might increase mortality	Severe or critical	NCT04403685
				Reduce oxygen requirement, ICU stay, median hospital stay and mortality	Critical	NCT04730323
				No better clinical status or lower mortality at 28 days	Severe	NCT04320615
				No prevention of intubation or death	Moderate	NCT04356937
**Repurposed drugs with immunomodulatory properties**	Thalidomide	Yes (3)	No result published			
	Isotretinoin	Yes (9)	No result published			
	Imatinib	Yes (5)	No result published			
	Atorvastatin	Yes (9)	No result published			
	Rosiglitazone	No	No clinical trial			
	Vemurafenib	No	No clinical trial			
**Immunosupressive drugs**	Tacrolimus	Yes (4)	No result published			
	Mycophenolate	No	No result published			
**Cytokines**	Interferon beta	Yes (13)	No result published			
**Anti-inflammatory drugs**	Parthenolid	No	No clinical trial			
	Curcumin	Yes (2)	No result published			
	Aspirin	yes (16)	No result published			

## Discussion

While immune characterization of the severe COVID-19 patients is now quite precise, notably dysregulated responses with a strong inflammation and a defect of IFN response (36, 37), a few immunologic studies on predictive factors of the clinical outcome and drug selection have been made (38, 39).

Different molecules of interest were pointed out that could be targeted in severe COVID-19 patients. A multi-omics analysis identified 219 molecules highly correlated with COVID-19 status and severity, involved in complement system activation, dysregulated lipid transport and neutrophil activation, vessel damage and blood coagulation (34). The chemokine CXCL10 has also been identified as a plasma biomarker of impaired CD4^+^ and CD8^+^ T cell responses in acute COVID-19 (22), and S100A8 and S100A9 alarmins, known to be released by myeloid cells in inflammatory situations, are biomarkers of monocytes and neutrophil subsets alterations (20). Finally, a recent study revealed that a combination of 12 biomarkers, including CCL2, IL-15, soluble ST2 (sST2), NGAL, sTNFRSF1A, ferritin, IL-6, S100A9, MMP-9, IL-2, sVEGFR1 and IL-10, was associated with mortality (38). As observed in previous studies (18, 21), our cohort of patients exhibit a profound lymphopenia and alterations of the myeloid compartment, with an increase of circulating “dysfunctional” CD14^+^CD163^+^ and CD14^+^HLA^-^DR^low^ monocytes as compared to controls. The immune gene signature shows an enrichment in innate myeloid immune profile, complement and coagulation cascades—consistent with the complement activation and hemostasis troubles described in COVID-19 (34)—and in neutrophil activation and degranulation—in line with neutrophils count and neutrophil extracellular traps (NETs) that were reported to be associated with COVID-19 severity (40). Altogether, these first results confirmed that despite a small number of patients, our cohort exhibited similar immune characteristics compared with previous published cohorts of severe COVID-19 patients.

The need of robust predictive biomarkers of COVID-19 severity led us to deeply characterize the immune signature of hospitalized COVID-19 patients at admission to search for early immune specificities that are linked to later multi-organ failure and severity of the disease. We have observed a strong heterogeneity among hospitalized COVID-19 patients and found two distinct groups. These two sub-groups of hospitalized Covid-19 patients were characterized by a distinct immune gene signature, and clinical outcome.

The group 1 is closer to the control group whereas the group 2 is more distant. Indeed, compared to group 1, the group 2 overexpresses a signature linked to myeloid immune response, cytokine mediated signaling pathways including type I IFN, and neutrophils chemotaxis and under-expresses a signature linked to NK and CD8^+^ T cell responses, T cell activation, Th17 response and antigen presenting pathways. Conflicting results have been reported for the role of type I IFN in COVID-19 patients: while type I IFNs are essential to control the disease in the early steps of infection, it seems to exacerbate inflammation during severe disease (41).

Interestingly, we also found an over-expression in the group 2 of alarmins (S100A8 and S100A12) that have been shown to be specifically linked with neutrophil recruitment in fatal coronavirus infections (18, 20, 42). Of note, if pro-inflammatory cytokines were correlated with monocytes clusters more abundant in COVID-19 patients, they were not different between groups 1 and 2. This is probably due to the small number of patients in each group.

Dramatically, when we compared the clinical data between the two groups, the group 2 was more severe than the group 1, showing a strong correlation between immune signatures and the severity of the disease. Indeed, the group 2 was characterized by a multi-organ failure (as indicated by the SOFA score) and an increased mortality rate: 44% of the group 2, and only 12% in the group 1. Of note, the only patient from group 1 who died suffered from recurrent breast cancer.

To assess whether segregation of hospitalized severe patients was still found in an independent larger cohort, we applied our immune signatures A and B on a public database of 100 hospitalized COVID-19 patients (GSE157103). Again, the patients expressing the signature A at admission were more often admitted in ICU, needed more frequently invasive mechanical ventilation and their comorbidity (Charlson) and severity (SOFA and APACHE II) scores were higher than patients expressing the signature B. This result confirms the strength of the correlation between our immune profiles and the clinical severity of the disease.

**Figure 6:**
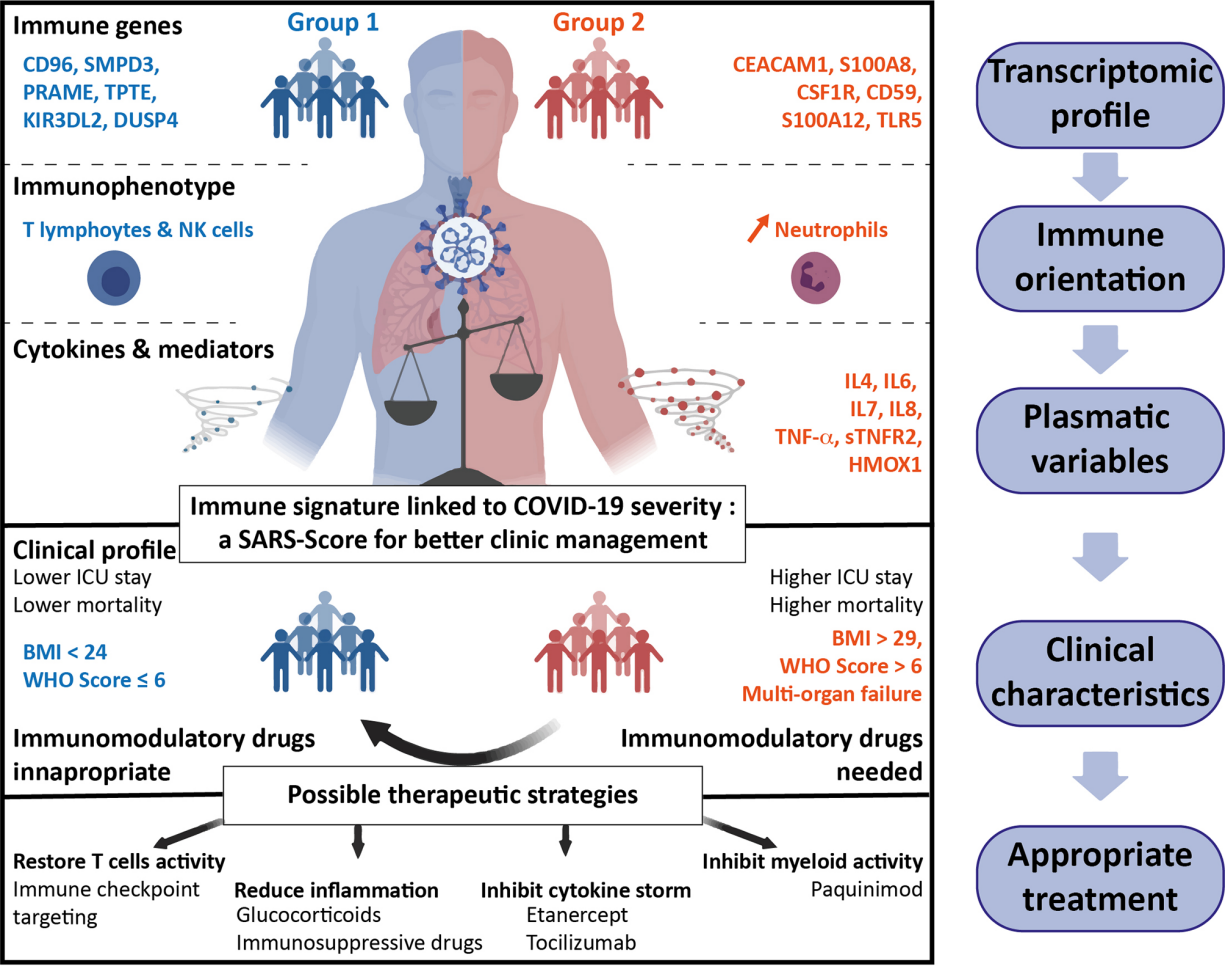
Summary of the two groups of hospitalized COVID-19 patients. Groups 1 (blue, left part) and 2 (red, right part) have been defined on differential immune transcriptomic profiles that correspond to specific immune orientations. The plasmatic and clinical characteristics of these two groups allow to predictively classify the patients and personalize the therapeutic strategies to improve the outcome of COVID-19 patients.

Finally, knowing that our immune signature could be predictive of the clinical outcome of COVID-19, we created a score, the SARS-Score, to classify the patients with easy obtainable clinical data and highly specific genes. We propose a score, composed of eight clinical parameters reflecting multi-organ failure and seven genes from our transcriptomic analysis, after their validation at the protein level. Applied to our cohort, the SARS-Score allows perfectly discriminating patients of groups 1 and 2, either using clinical data and/or immune variables, and even allows proposing a therapy specific to the group the patient belongs to (**Figure 6**).

Indeed, results about treatments of COVID-19 patients are highly controversial (43–46), probably in part because severe COVID-19 patients were considered as a homogeneous population. Based on the immune signature, we performed a drug gene set enrichment analysis to propose differential therapeutics, alone or in combination, that could specifically correspond to each group of patients. With this approach, we took into account both the specific pharmacology of the drug and the overall effect on the modulation of genes expression. We found several drugs that would revert the signatures A and B: our study suggests that inhibiting pro-inflammatory cytokines would be beneficial for group 2 patients. This could be achieved by cytokine receptors blockade such as Tocilizumab or Etanercept to target IL-6R or TNFαR, respectively. Blocking TNFαR has already been proposed in COVID-19 (47) and might be a strong candidate as it can regulate both signatures A and B. On the contrary, group 1 patients would probably benefit from drugs that activate antiviral adaptive immune cell responses. This could be achieved by repurposing molecules such as Imatinib, Thalidomide, Isotretinoin or Atorvastatin, that are already in clinical trials in COVID-19 (NCT04422678, NCT04273529, NCT04361422 and NCT04380402. respectively) (30, 48–50), or monoclonal antibodies targeting immune co-stimulatory molecules TNFRSF4 (OX40), TNFRSF18 (GITR) or LAG3 immune checkpoint (overexpressed in group 1 patients). Immunosuppressive drugs like Tacrolimus—in clinical trials in COVID-19 (NCT04341038)—appear also as a possible candidate in our study, although its use has been associated with high mortality among organ transplant recipients. Finally, glucocorticoids such as Dexamethasone, Prednisolone or Hydrocortisone seem to be able to both boost signature B profile and revert signature A. Interestingly, the clinical efficacy of corticosteroids have already been correlated with neutrophil-to-lymphocytes ratio, which we found as a marker of group 2 patients (51, 52).

Altogether, our study provides a fundamental understanding of the different immune profiles among severe hospitalized COVID-19 patients and provides a score which would be a useful tool to classify patients and propose accurate treatments for both groups of patients, towards a more personalized medicine against COVID-19.

## Data Availability Statement

The raw data supporting the conclusions of this article will be made available by the authors, without undue reservation.

## Ethics Statement

The studies involving human participants were reviewed and approved by CERAPHP·5 00011928. The patients/participants provided their written informed consent to participate in this study.

## Author Contributions

JR, PEF and NJ performed the experiments. JR, PEF, AKi and MM performed bioinformatic analysis. JR, P-EF, NJ, and IC analyzed the data. NH, AKa, CS, ET, DS, AK, J-SH,ML, AF, JBA, JLD,MADD, and FP provided clinical samples and pathological data. IC designed and supervised the study. JR, PEF, NJ, MADD, FP and IC wrote the manuscript. All authors contributed to the article and approved the submitted version.

## Funding

This work was supported by the “Institut National de la Sante et de la Recherche Medicale” (INSERM), Sorbonne Universite, Universite de Paris.

## Conflict of Interest

The authors declare that the research was conducted in the absence of any commercial or financial relationships that could be construed as a potential conflict of interest.
