# Supplementary material for: Immune Signature Linked to COVID-19 Severity: A SARS-Score for Personalized Medicine
Source: Front Immunol. 2021 Jul 12;12:701273. doi: 10.3389/fimmu.2021.701273 (PMC8312547; doi:10.3389/fimmu.2021.701273)
Supplement: Supplementary file 1 [file DataSheet_1.docx]

**Supplemental Material**

**
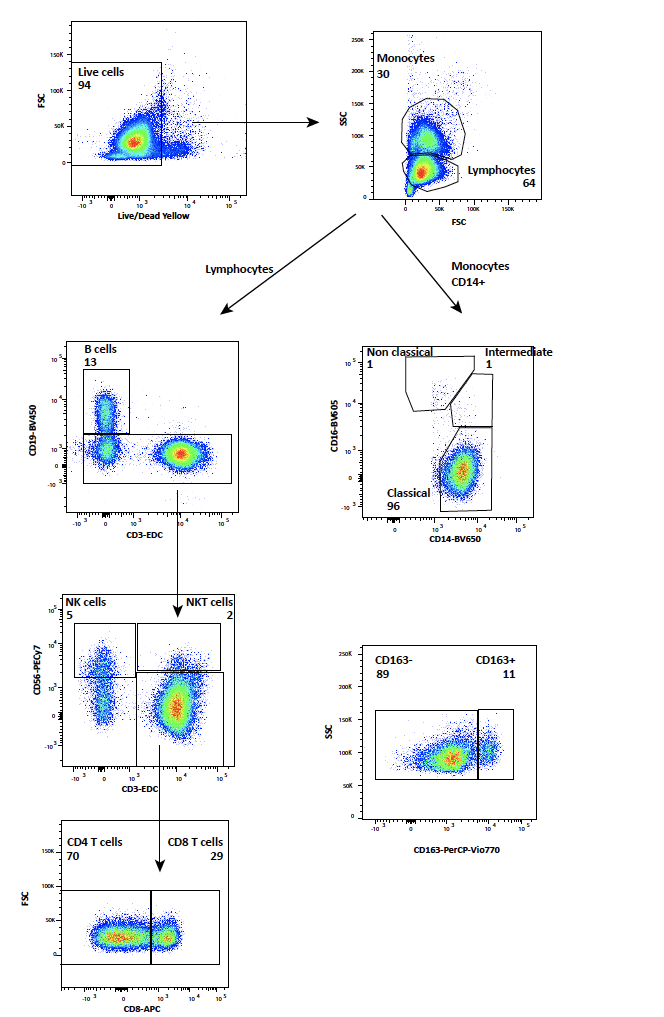
**

**Supplementary Figure 1. Flow cytometry gating strategy for PBMCs.** Among live cells, monocytes were defined as “SSC High” and lymphocytes as “SSC Low”. Among lymphocytes, B cells were characterized as CD3^-^CD19^+^, NK cells as CD19^-^CD3^-^CD56^+^, NKT cells as CD19^-^CD3^+^CD56^+^, T lymphocytes as CD19^-^CD3^+^ CD4^+^ or CD8^+^. The sub-populations of monocytes were defined according to their expression of CD14 and CD16 (classical/intermediate/non-classical monocytes) or CD163 (CD163^-^/CD163^+^).

**
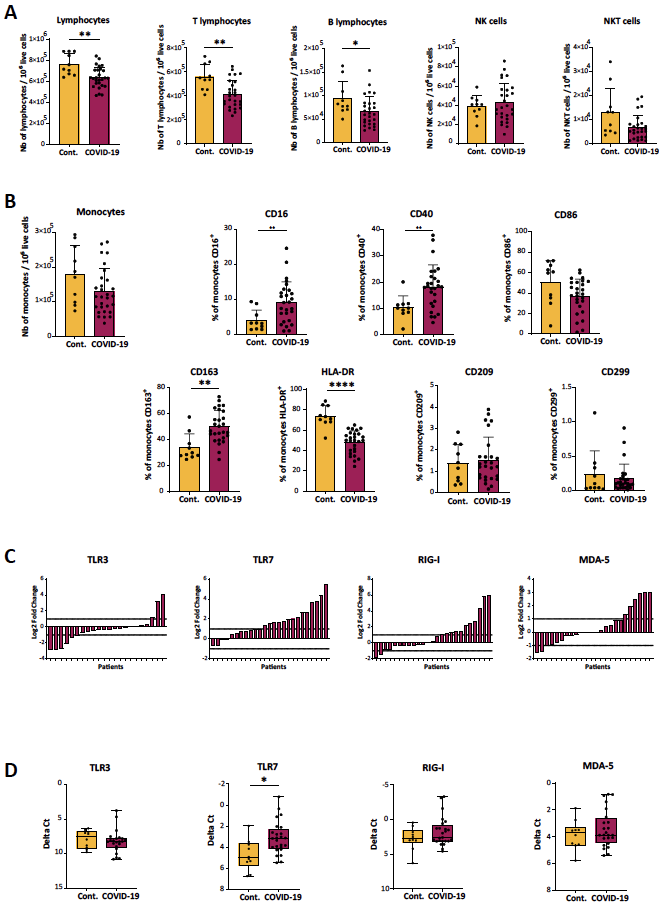
**

**Supplementary Figure 2. Comparison of PBMC from healthy donors and COVID-19 patients.** (**A**) Lymphocytes, NK and NKT cells and (**B**) monocytes were quantified by flow cytometry in healthy donors (“Cont.”, yellow) and COVID-19 patients (red). (**C**) Gene expression of TLR3, TLR7, RIG-I and MDA-5 was evaluated by real time PCR. COVID-19 patients are classified from the lowest to the highest, according to the log2 fold change in comparison to the mean expression of each receptors of healthy donors. **(D)** Box plot representation of delta CT of each pattern recognition receptors for healthy donors (“Cont.”, yellow) and COVID-19 patients (red). Statistical analyses were performed by Wilcoxon test using GraphPad software. *: p<0.05; **: p<0.01.


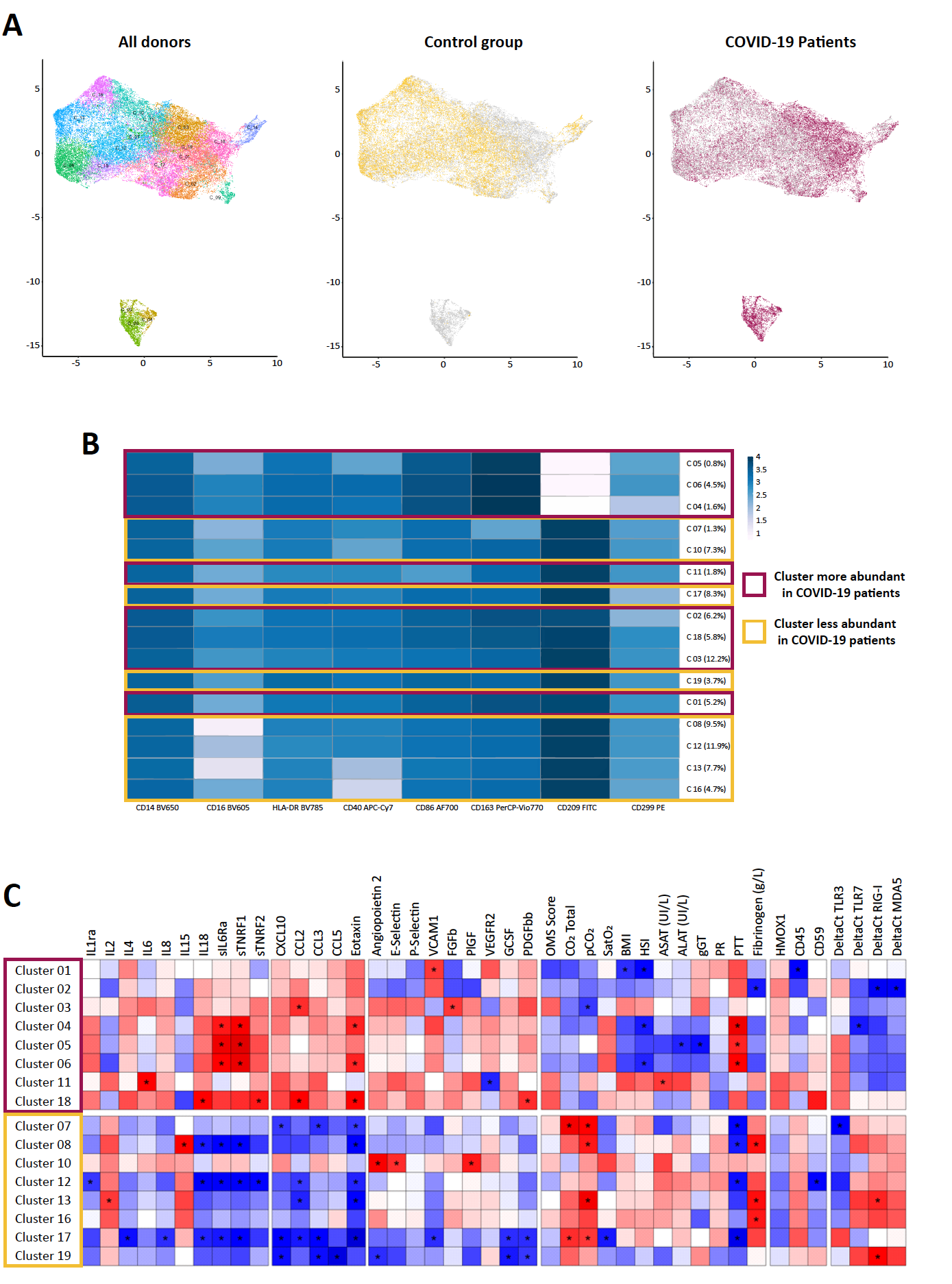


**Supplementary Figure 3. Unsupervised analysis of flow cytometry data to compare healthy donors and COVID-19 patients.** (**A**) Uniform Manifold Approximation and Projection (UMAP) of CD14^+^ cells from all samples (left panel), healthy donors (central panel) and COVID-19 patients (right panel). (**B**) Heatmap showing the phenotype of the 8 clusters up-regulated (surrounded in purple) or down-regulated (surrounded in yellow) in COVID-19 patients. (**C**) Correlation matrix showing the spearman correlation between the clusters (differentially abundant between healthy donors and COVID patients) and cytokine and chemokines production, endothelium linked-molecules and clinical data. Significant correlations (p-values < 0.05) are indicated with a star.


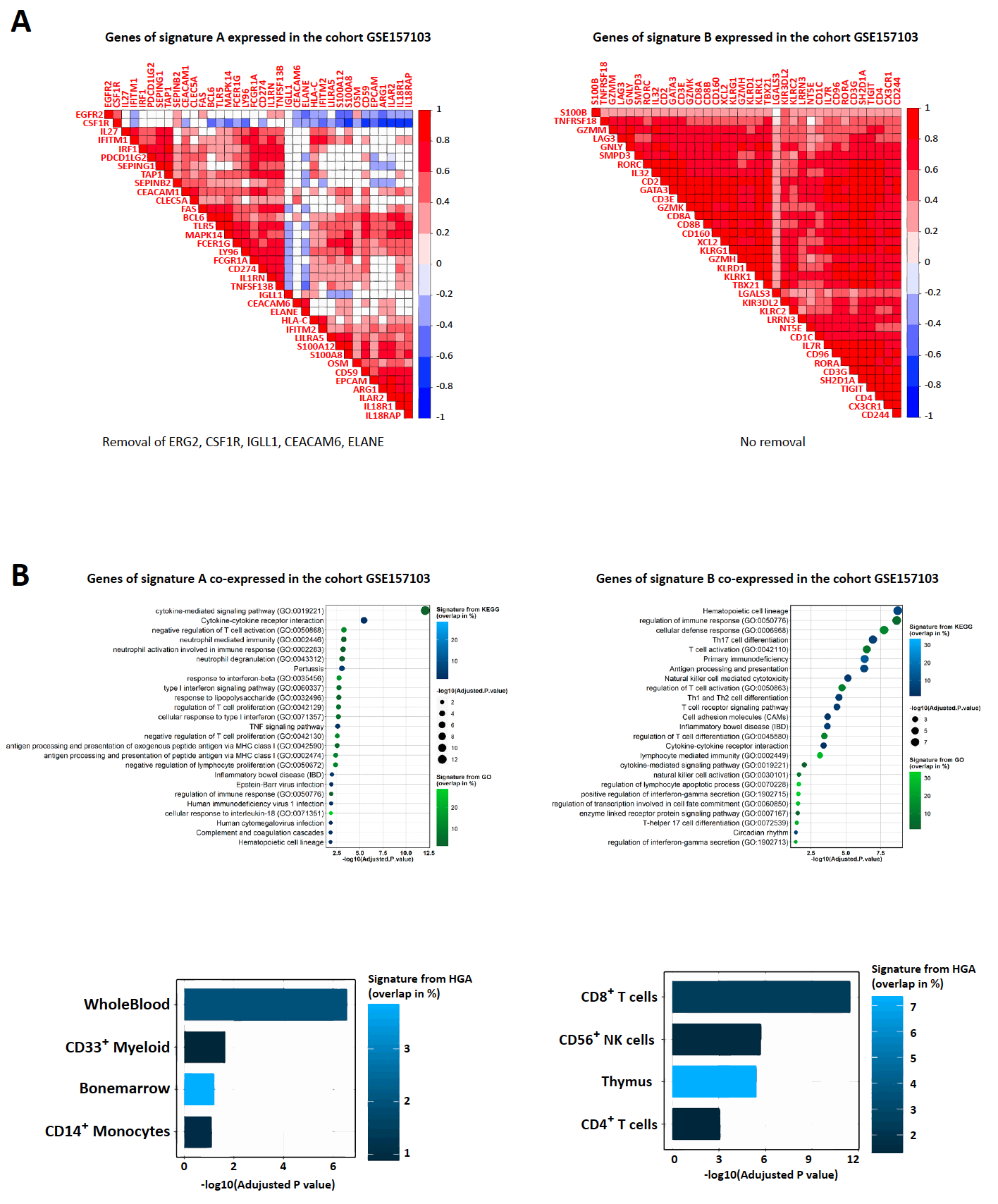


**Supplementary figure 4.** **Expression of signatures A and B in a public RNAseq cohort. (A)** Correlation between genes with TPM < 1 belonging to signatures A and B respectively in the public RNA-seq cohort (GSE157103). Genes that are not co-expressed are eliminated from the signature. **(B)** Enrichment analysis of the signatures A and B.

**
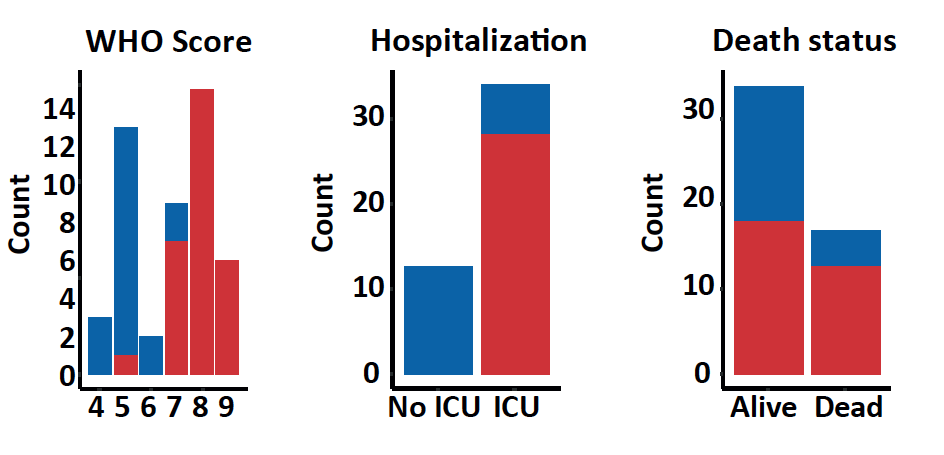
**

**Supplementary figure 5.** Comparison of WHO Score, type of hospitalization and death status between group 1 (blue) and 2 (red) COVID-19 patients.

**Supplementary table 1: Clinical description of the COVID-19 patient cohort.**

| **Patient ID** | **Age** | **Gender** | **Diabetes** | **Arterial hypertension** | **BMI** | **ICU Admission** | **Death** |
| --- | --- | --- | --- | --- | --- | --- | --- |
| 1 | 53 | M | No | No | 19.7 | No | No |
| 2 | 57 | M | No | No | 26.4 | Yes | No |
| 3 | 57 | M | No | No | 28.0 | Yes | No |
| 4 | 77 | F | No | No | 22.6 | Yes | Yes |
| 5 | 52 | M | No | Yes | 25.5 | Yes | No |
| 6 | 60 | M | Yes | Yes | 24.5 | No | No |
| 7 | 34 | F | No | No | 27.0 | No | No |
| 8 | 48 | M | No | No | 25.4 | Yes | No |
| 9 | 70 | M | Yes | Yes | 24.0 | Yes | Yes |
| 10 | 63 | F | No | No | 28.3 | Yes | Yes |
| 11 | 35 | M | No | No | 31.0 | Yes | Yes |
| 12 | 53 | M | No | Yes | 26.8 | Yes | No |
| 13 | 44 | M | Yes | No | 32.0 | Yes | No |
| 14 | 73 | M | No | Yes | 36.0 | Yes | Yes |
| 15 | 59 | F | No | Yes | 26.3 | Yes | Yes |
| 16 | 75 | M | No | No | 29.2 | Yes | No |
| 17 | 54 | F | No | Yes | 27.8 | Yes | No |
| 18 | 85 | M | No | Yes | 23.2 | Yes | Yes |
| 19 | 59 | F | No | No | 36.7 | Yes | No |
| 20 | 68 | F | Yes | Yes | 42.0 | Yes | No |
| 21 | 64 | M | No | Yes | 24.2 | Yes | No |
| 22 | 75 | M | No | Yes | 28.4 | Yes | Yes |
| 23 | 51 | M | No | Yes | 21.9 | Yes | No |
| 24 | 53 | M | No | No | 29.9 | Yes | No |
| 25 | 78 | F | Yes | No | 30.1 | No | Yes |
| 26 | 87 | M | No | Yes | 24.0 | No | Yes |
| 27 | 84 | M | No | No | 27.1 | Yes | No |
| 28 | 74 | F | Yes | Yes | 29.2 | No | No |
| 29 | 71 | M | No | No | 26.0 | Yes | No |
| 30 | 53 | M | Yes | Yes | 33.0 | Yes | Yes |
| 31 | 55 | M | No | No | 24.4 | Yes | No |
| 32 | 53 | M | No | Yes | 38.0 | Yes | No |
| 33 | 65 | M | No | Yes | 21.6 | Yes | Yes |
| 34 | 63 | M | Yes | Yes | 25.8 | No | No |
| 35 | 65 | M | No | No | 25.0 | No | No |
| 36 | 58 | F | Yes | Yes | 29.8 | No | No |

**Supplementary table 2: List of up-regulated genes in COVID-19 patients vs healthy donors**

List of the up-regulated genes in COVID-19 patients compared to the control group. The genes are classified by the log2 fold change (Log FC) and the adjusted p-value (Q-value) is also displayed for each gene. The thresholds used to define up-regulated genes are a Q-values < 0.05 (adjusted p-values) and a fold change > 2 (log2 fold change > 1).

| **Names** | **Log**  **FC** | **Q-values** | **Names** | **Log**  **FC** | **Q-values** | **Names** | **Log**  **FC** | **Q-values** |
| --- | --- | --- | --- | --- | --- | --- | --- | --- |
| **SPACA3** | 36.34 | 3.55E-03 | **PRG2** | 2.05 | 4.44E-04 | **SLC11A1** | 1.42 | 2.10E-08 |
| **FN1** | 31.62 | 5.93E-08 | **C2** | 2.03 | 6.97E-04 | **RAG1** | 1.41 | 3.43E-05 |
| **PRAME** | 30.15 | 2.79E-02 | **MX1** | 2.01 | 1.79E-04 | **SPINK5** | 1.41 | 1.63E-02 |
| **PBK** | 29.38 | 7.01E-11 | **TTK** | 2.00 | 1.23E-07 | **SERPINB2** | 1.40 | 9.74E-06 |
| **TREM2** | 26.48 | 1.56E-03 | **IL18RAP** | 1.99 | 4.47E-08 | **CD58** | 1.39 | 2.65E-13 |
| **RRAD** | 24.11 | 3.78E-09 | **ELANE** | 1.97 | 3.76E-05 | **MYD88** | 1.38 | 4.77E-15 |
| **AXL** | 22.76 | 2.96E-03 | **CSF3** | 1.95 | 1.99E-06 | **EGR2** | 1.37 | 5.34E-05 |
| **CFB** | 6.70 | 4.27E-05 | **LY96** | 1.94 | 2.38E-11 | **CCL18** | 1.37 | 1.08E-03 |
| **IFI27** | 6.21 | 7.80E-13 | **IL24** | 1.93 | 9.92E-08 | **SAA1** | 1.36 | 5.63E-04 |
| **PPARG** | 5.86 | 1.90E-13 | **XCR1** | 1.93 | 2.40E-09 | **JAK2** | 1.36 | 1.05E-13 |
| **CTSG** | 4.94 | 1.10E-03 | **ITGB4** | 1.91 | 6.24E-03 | **FCGR2A** | 1.35 | 8.64E-09 |
| **CXCL11** | 4.55 | 9.62E-05 | **IFIH1** | 1.90 | 1.30E-07 | **CKLF** | 1.32 | 4.00E-08 |
| **ARG1** | 4.02 | 4.34E-10 | **CD24** | 1.90 | 5.35E-04 | **STAT1** | 1.32 | 6.16E-08 |
| **CDK1** | 3.97 | 0.00E+00 | **CCR1** | 1.89 | 2.37E-08 | **JAK3** | 1.31 | 3.17E-10 |
| **MPPED1** | 3.81 | 3.36E-07 | **OSM** | 1.89 | 1.02E-07 | **CTSL** | 1.31 | 1.19E-04 |
| **LCN2** | 3.76 | 7.08E-08 | **TNFSF13B** | 1.87 | 2.13E-08 | **NOD2** | 1.30 | 2.72E-05 |
| **CCL27** | 3.75 | 1.16E-08 | **TLR2** | 1.85 | 0.00E+00 | **CXCL2** | 1.29 | 4.25E-05 |
| **LTF** | 3.75 | 4.67E-07 | **ITGA2B** | 1.85 | 1.06E-07 | **FPR2** | 1.28 | 1.76E-09 |
| **CEACAM8** | 3.68 | 3.20E-06 | **IFI35** | 1.84 | 4.96E-08 | **TLR1** | 1.27 | 1.33E-10 |
| **SIGLEC1** | 3.67 | 3.36E-06 | **FCER1G** | 1.82 | 1.51E-11 | **IL27** | 1.26 | 2.69E-05 |
| **CEACAM6** | 3.61 | 7.62E-07 | **LILRA5** | 1.82 | 8.91E-10 | **MARCO** | 1.25 | 9.51E-05 |
| **CCL1** | 3.59 | 1.03E-10 | **F12** | 1.81 | 3.45E-10 | **THY1** | 1.25 | 1.85E-03 |
| **SERPING1** | 3.36 | 8.01E-10 | **C6** | 1.81 | 1.38E-03 | **CEBPB** | 1.24 | 1.55E-07 |
| **BIRC5** | 3.24 | 0.00E+00 | **CD70** | 1.80 | 2.32E-09 | **C1QA** | 1.21 | 1.06E-02 |
| **SSX4** | 3.23 | 1.58E-04 | **DDX58** | 1.79 | 8.94E-08 | **CREB5** | 1.21 | 2.74E-07 |
| **CHIT1** | 3.16 | 2.07E-10 | **MUC1** | 1.78 | 5.29E-08 | **IL34** | 1.21 | 1.10E-02 |
| **S100A7** | 3.11 | 3.45E-06 | **IFIT2** | 1.78 | 1.08E-03 | **ICAM1** | 1.21 | 1.20E-09 |
| **S100A12** | 3.10 | 8.37E-13 | **MAGEA3** | 1.76 | 1.30E-03 | **SELL** | 1.20 | 5.91E-07 |
| **CD274** | 3.10 | 9.95E-14 | **CCL8** | 1.74 | 4.51E-02 | **FAS** | 1.19 | 1.86E-06 |
| **C3AR1** | 3.09 | 1.49E-13 | **IL1R1** | 1.73 | 5.33E-06 | **PTPRC** | 1.17 | 2.71E-07 |
| **CCL13** | 3.04 | 5.13E-11 | **CD38** | 1.69 | 2.28E-08 | **ADORA2A** | 1.17 | 7.28E-07 |
| **ISG15** | 3.02 | 7.17E-06 | **VEGFC** | 1.69 | 1.50E-04 | **TLR4** | 1.17 | 2.81E-06 |
| **FCGR1A** | 3.01 | 1.05E-13 | **ITGA1** | 1.68 | 0.00E+00 | **C9** | 1.16 | 9.50E-04 |
| **CLEC5A** | 2.94 | 1.62E-14 | **IL25** | 1.67 | 9.57E-04 | **A2M** | 1.16 | 2.83E-02 |
| **CXCL10** | 2.93 | 1.19E-06 | **BATF** | 1.67 | 5.52E-12 | **FCGR3A** | 1.15 | 8.37E-04 |
| **IFIT1** | 2.91 | 2.51E-05 | **IFNL2** | 1.66 | 3.91E-05 | **IFNL1** | 1.15 | 1.01E-04 |
| **CRP** | 2.86 | 1.11E-06 | **TNFSF15** | 1.64 | 2.23E-04 | **TNFRSF13B** | 1.13 | 3.80E-03 |
| **IL10** | 2.86 | 1.78E-06 | **POU2AF1** | 1.64 | 9.84E-06 | **TLR10** | 1.13 | 1.76E-09 |
| **OAS3** | 2.81 | 2.82E-06 | **IL1B** | 1.63 | 3.95E-08 | **TLR8** | 1.12 | 5.25E-07 |
| **PDCD1LG2** | 2.75 | 7.86E-10 | **CCRL2** | 1.62 | 6.40E-07 | **PRKCD** | 1.12 | 1.22E-12 |
| **TNFRSF17** | 2.73 | 1.07E-08 | **ITGAM** | 1.61 | 0.00E+00 | **IL17RA** | 1.11 | 3.81E-10 |
| **TLR5** | 2.72 | 0.00E+00 | **IRAK2** | 1.59 | 4.28E-09 | **CD164** | 1.11 | 2.46E-11 |
| **S100A8** | 2.68 | 8.53E-15 | **TAP1** | 1.56 | 1.20E-09 | **CDKN1A** | 1.10 | 3.34E-09 |
| **CAMP** | 2.66 | 1.90E-06 | **IL4R** | 1.54 | 9.23E-09 | **BST2** | 1.08 | 1.55E-04 |
| **IL1R2** | 2.55 | 1.06E-07 | **IRF4** | 1.54 | 6.84E-05 | **NCF4** | 1.08 | 1.03E-07 |
| **PLAU** | 2.46 | 0.00E+00 | **ARG2** | 1.52 | 6.48E-06 | **SPA17** | 1.08 | 9.29E-07 |
| **IFITM1** | 2.39 | 2.65E-13 | **CASP1** | 1.51 | 4.77E-15 | **SBNO2** | 1.08 | 1.04E-07 |
| **MAPK14** | 2.39 | 0.00E+00 | **CD59** | 1.50 | 5.17E-07 | **CLEC4A** | 1.08 | 6.47E-08 |
| **CEACAM1** | 2.37 | 8.21E-10 | **NFKBIA** | 1.50 | 2.89E-13 | **CSF1R** | 1.07 | 6.88E-06 |
| **IL18R1** | 2.37 | 3.16E-10 | **MERTK** | 1.47 | 4.59E-06 | **IFNGR1** | 1.05 | 1.82E-09 |
| **CTAG1B** | 2.28 | 1.17E-08 | **CD63** | 1.47 | 1.06E-12 | **ITGAX** | 1.05 | 3.38E-07 |
| **IRF7** | 2.27 | 7.59E-08 | **BST1** | 1.47 | 0.00E+00 | **CD14** | 1.04 | 3.17E-10 |
| **CR1** | 2.25 | 2.42E-12 | **IL12A** | 1.46 | 4.76E-07 | **STAT2** | 1.04 | 2.91E-04 |
| **C1QB** | 2.25 | 2.79E-08 | **ITGB3** | 1.45 | 1.96E-05 | **TNFSF14** | 1.04 | 1.04E-07 |
| **BCL6** | 2.22 | 5.16E-11 | **IFITM2** | 1.45 | 7.51E-08 | **ENTPD1** | 1.03 | 8.34E-08 |
| **LAMP3** | 2.22 | 4.39E-06 | **IFI16** | 1.44 | 1.28E-08 | **PSMB9** | 1.02 | 6.29E-07 |
| **CXCL3** | 2.22 | 4.43E-08 | **SOCS1** | 1.44 | 1.16E-05 | **TLR6** | 1.01 | 5.82E-09 |
| **IL1RN** | 2.09 | 1.81E-08 | **FLT3** | 1.43 | 9.84E-05 | **CD207** | 1.00 | 5.54E-03 |
| **FUT7** | 2.06 | 0.00E+00 | **PDGFC** | 1.43 | 5.59E-07 |  |  |  |
| **ULBP2** | 2.06 | 1.25E-05 | **TNFSF10** | 1.42 | 6.65E-09 |  |  |  |

**Supplemental table 3: List of down-regulated genes in COVID-19 patients vs healthy donors**

List of the down-regulated genes in COVID-19 patients compared to the control group. The genes are classified by the log2 fold change (Log FC) and the adjusted p-value (Q-value) is also displayed for each gene. The thresholds used to define down-regulated genes are a Q-values < 0.05 (adjusted p-values) and a fold change < -2 (log2 fold change < -1).

| **Names** | **logFC** | **Q-values** | **Names** | **logFC** | **Q-values** |
| --- | --- | --- | --- | --- | --- |
| **FOXJ1** | -27.90 | 8.07E-03 | **CD96** | -1.37 | 3.08E-10 |
| **CD209** | -24.51 | 1.76E-03 | **KLRG1** | -1.35 | 1.10E-04 |
| **IL6** | -21.63 | 6.82E-03 | **IL21R** | -1.34 | 4.21E-11 |
| **CCL24** | -3.70 | 3.11E-02 | **GZMK** | -1.34 | 3.03E-04 |
| **FCER1A** | -3.52 | 0.00E+00 | **HLA-DPA1** | -1.32 | 4.25E-08 |
| **IL4** | -3.47 | 0.00E+00 | **CD6** | -1.32 | 4.26E-09 |
| **LRRN3** | -2.58 | 3.90E-09 | **CR2** | -1.29 | 9.56E-08 |
| **IL23R** | -2.42 | 8.50E-13 | **RORA** | -1.28 | 5.64E-09 |
| **LILRA4** | -2.40 | 0.00E+00 | **IL12RB2** | -1.27 | 1.02E-07 |
| **MS4A2** | -2.33 | 0.00E+00 | **KIR2DS1** | -1.26 | 1.14E-02 |
| **KLRB1** | -2.33 | 0.00E+00 | **ETS1** | -1.25 | 4.70E-10 |
| **KIT** | -2.16 | 1.21E-05 | **KLRK1** | -1.23 | 1.15E-04 |
| **SPP1** | -2.07 | 5.86E-03 | **CD3E** | -1.22 | 4.44E-07 |
| **RORC** | -2.06 | 9.83E-12 | **CXCR5** | -1.22 | 5.70E-09 |
| **KIR3DL2** | -1.91 | 3.59E-04 | **KLRC1** | -1.22 | 4.12E-05 |
| **CD1C** | -1.87 | 1.21E-11 | **LY9** | -1.20 | 7.68E-10 |
| **PTGDR2** | -1.83 | 1.13E-04 | **IL11RA** | -1.19 | 2.13E-13 |
| **CD160** | -1.79 | 3.79E-03 | **XCL2** | -1.19 | 5.20E-04 |
| **TCF7** | -1.71 | 5.02E-14 | **DOCK9** | -1.19 | 7.20E-13 |
| **DUSP4** | -1.64 | 2.01E-03 | **NEFL** | -1.18 | 9.55E-03 |
| **ABCB1** | -1.63 | 2.37E-12 | **CD244** | -1.17 | 1.45E-06 |
| **SH2D1B** | -1.58 | 7.03E-10 | **FCER2** | -1.16 | 4.67E-05 |
| **CD4** | -1.57 | 1.13E-09 | **CD247** | -1.15 | 1.07E-06 |
| **DPP4** | -1.56 | 7.03E-13 | **CARD11** | -1.15 | 1.79E-08 |
| **TNFRSF11A** | -1.55 | 1.57E-02 | **NFATC2** | -1.15 | 2.02E-08 |
| **LTK** | -1.55 | 1.81E-10 | **ICOSLG** | -1.14 | 1.23E-07 |
| **GATA3** | -1.52 | 5.18E-08 | **SH2D1A** | -1.14 | 2.35E-06 |
| **TIGIT** | -1.51 | 1.32E-08 | **LCK** | -1.14 | 5.65E-08 |
| **IL2RB** | -1.51 | 7.07E-13 | **KLRC2** | -1.14 | 3.95E-03 |
| **CCR3** | -1.51 | 6.48E-06 | **ICOS** | -1.13 | 9.48E-09 |
| **CLEC4C** | -1.50 | 5.86E-03 | **FLT3LG** | -1.12 | 1.62E-09 |
| **MAGEB2** | -1.48 | 7.32E-05 | **SIGIRR** | -1.12 | 4.26E-09 |
| **CD40LG** | -1.43 | 5.30E-11 | **TNFRSF18** | -1.09 | 3.95E-04 |
| **CD7** | -1.43 | 1.49E-12 | **CD3G** | -1.07 | 7.69E-05 |
| **CD5** | -1.41 | 1.34E-09 | **HLA-DRB3** | -1.07 | 2.00E-06 |
| **CCR7** | -1.40 | 1.62E-08 | **ZAP70** | -1.05 | 1.58E-06 |
| **HLA-DPB1** | -1.39 | 9.84E-09 | **SPN** | -1.04 | 1.36E-09 |
| **NT5E** | -1.39 | 2.59E-05 | **CD1A** | -1.04 | 1.16E-03 |
| **CD28** | -1.39 | 7.07E-13 | **RUNX3** | -1.04 | 1.08E-07 |
| **IL7R** | -1.38 | 3.27E-07 | **HLA-DRA** | -1.02 | 1.25E-05 |

**Supplemental table 4: List of up-regulated genes in group 2 vs group 1 (signature A)**

List of the up-regulated genes in group 2 patients compared to the group 1. The genes are classified by the log2 fold change (Log FC) and the adjusted p-value (Q-value) is also displayed for each gene. The thresholds used to define up-regulated genes are a Q-values < 0.05 (adjusted p-values) and a fold change > 2 (log2 fold change > 1).

| **Names** | **logFC** | **Q-values** | **Names** | **logFC** | **Q-values** |
| --- | --- | --- | --- | --- | --- |
| **IL17A** | 29.19 | 4.10E-03 | **IL1R2** | 1.43 | 9.02E-03 |
| **TREM2** | 23.46 | 3.14E-03 | **IL25** | 1.38 | 2.92E-03 |
| **IL12B** | 5.74 | 7.41E-03 | **EGR2** | 1.37 | 9.06E-06 |
| **IFNA1** | 3.12 | 9.71E-03 | **IL27** | 1.35 | 5.21E-05 |
| **SSX4** | 2.78 | 6.99E-05 | **BCL6** | 1.34 | 5.68E-06 |
| **SPINK5** | 2.18 | 1.23E-03 | **CD274** | 1.34 | 1.75E-04 |
| **MAGEA3** | 2.17 | 1.33E-04 | **IL1RN** | 1.32 | 2.22E-04 |
| **MPPED1** | 2.14 | 2.14E-05 | **IL1RAPL2** | 1.31 | 5.37E-04 |
| **ARG1** | 2.08 | 6.29E-04 | **IFNL2** | 1.26 | 1.32E-03 |
| **CD34** | 1.87 | 3.14E-03 | **TNFSF13B** | 1.25 | 2.53E-04 |
| **TPSAB1** | 1.84 | 8.11E-04 | **CSF1R** | 1.23 | 3.63E-10 |
| **SERPING1** | 1.83 | 2.09E-04 | **CD59** | 1.23 | 1.64E-05 |
| **S100A12** | 1.83 | 4.29E-08 | **MRC1** | 1.22 | 2.10E-03 |
| **CCL1** | 1.81 | 2.35E-06 | **LILRA5** | 1.19 | 7.75E-06 |
| **IGLL1** | 1.8 | 5.71E-03 | **IL18R1** | 1.19 | 2.33E-03 |
| **C8B** | 1.79 | 2.56E-02 | **IFITM1** | 1.19 | 5.10E-05 |
| **ULBP2** | 1.74 | 5.38E-05 | **TLR5** | 1.18 | 8.52E-07 |
| **PDCD1LG2** | 1.73 | 1.82E-05 | **FCER1G** | 1.18 | 1.28E-07 |
| **CCR9** | 1.72 | 6.97E-05 | **IFNA7** | 1.16 | 1.90E-02 |
| **CCL27** | 1.7 | 9.57E-05 | **IL18RAP** | 1.14 | 3.11E-03 |
| **ROPN1** | 1.68 | 3.81E-04 | **SERPINB2** | 1.13 | 6.94E-04 |
| **RRAD** | 1.65 | 9.01E-03 | **FAS** | 1.12 | 9.57E-07 |
| **CEACAM6** | 1.63 | 4.24E-02 | **MAPK14** | 1.09 | 3.61E-06 |
| **CTAG1B** | 1.62 | 4.93E-06 | **HLA-C** | 1.09 | 8.28E-04 |
| **CEACAM1** | 1.6 | 8.52E-07 | **CD276** | 1.07 | 1.58E-02 |
| **ELANE** | 1.6 | 1.36E-03 | **EPCAM** | 1.06 | 3.08E-02 |
| **CCL13** | 1.59 | 1.26E-06 | **CXCL2** | 1.05 | 3.29E-03 |
| **OSM** | 1.54 | 2.92E-06 | **RAG1** | 1.05 | 5.48E-03 |
| **CLEC5A** | 1.54 | 5.68E-06 | **IFITM2** | 1.03 | 5.68E-06 |
| **CRP** | 1.51 | 8.46E-04 | **PASD1** | 1.02 | 1.08E-02 |
| **FCGR1A** | 1.51 | 2.64E-06 | **IRF1** | 1.01 | 9.24E-07 |
| **CSF3** | 1.49 | 4.65E-05 | **TAP1** | 1.01 | 7.76E-05 |
| **IL34** | 1.49 | 2.50E-03 | **CT45A1** | 1.01 | 6.36E-03 |
| **S100A8** | 1.47 | 2.19E-12 | **LY96** | 1.01 | 9.55E-06 |
| **CCL18** | 1.43 | 2.48E-03 | **SAA1** | 1 | 2.76E-02 |

**Supplemental table 5: List of down-regulated genes in group 2 vs group 1 (Signature B)**

List of the down-regulated genes in group 2 patients compared to group 1. The genes are classified by the log2 fold change (Log FC) and the adjusted p-value (Q-value) is also displayed for each gene. The thresholds used to define down-regulated genes are a Q-values < 0.05 (adjusted p-values) and a fold change < -2 (log2 fold change < -1).

| **Names** | **logFC** | **Q-values** | **Names** | **logFC** | **Q-values** |
| --- | --- | --- | --- | --- | --- |
| **KIT** | -30.98 | 6.33E-03 | **GZMH** | -1.28 | 4.48E-03 |
| **TNFRSF11A** | -30.57 | 1.32E-03 | **CD8B** | -1.26 | 3.08E-04 |
| **TPTE** | -26.99 | 1.31E-02 | **KLRC2** | -1.25 | 5.29E-03 |
| **SMPD3** | -26.83 | 2.05E-04 | **LGALS3** | -1.23 | 4.04E-03 |
| **PRAME** | -25.98 | 1.71E-03 | **TNFRSF18** | -1.18 | 1.36E-02 |
| **KIR3DL2** | -25.37 | 1.91E-02 | **TIGIT** | -1.17 | 6.69E-06 |
| **DUSP4** | -24.82 | 2.61E-02 | **NT5E** | -1.16 | 1.64E-02 |
| **CD160** | -2.45 | 8.34E-04 | **CD96** | -1.16 | 2.19E-12 |
| **S100B** | -2.4 | 1.93E-02 | **IL32** | -1.14 | 5.97E-06 |
| **LRRN3** | -2.04 | 6.88E-05 | **ICOSLG** | -1.13 | 1.52E-06 |
| **GATA3** | -1.97 | 7.79E-03 | **CD3E** | -1.12 | 2.43E-06 |
| **CD8A** | -1.64 | 1.17E-04 | **SH2D1A** | -1.11 | 5.76E-07 |
| **RORC** | -1.62 | 8.72E-03 | **CD1C** | -1.09 | 1.53E-03 |
| **XCL2** | -1.55 | 5.71E-06 | **GZMM** | -1.09 | 5.76E-07 |
| **GZMK** | -1.52 | 3.08E-04 | **CX3CR1** | -1.09 | 8.89E-04 |
| **KLRK1** | -1.52 | 8.52E-07 | **KLRG1** | -1.08 | 1.30E-02 |
| **ITGB4** | -1.52 | 2.56E-02 | **CD244** | -1.07 | 3.61E-06 |
| **LAG3** | -1.48 | 1.91E-03 | **CD2** | -1.06 | 3.43E-04 |
| **KLRD1** | -1.44 | 4.79E-05 | **RORA** | -1.03 | 8.99E-07 |
| **GNLY** | -1.41 | 4.87E-05 | **CD4** | -1.02 | 4.73E-05 |
| **CD3G** | -1.32 | 8.52E-07 | **IL7R** | -1.02 | 1.59E-03 |
| **DDX43** | -1.31 | 2.65E-02 | **TBX21** | -1.01 | 9.59E-04 |
| **TNFRSF4** | -1.28 | 3.92E-04 |  |  |  |

**Supplemental table 6: Clinical comparison of groups 1 and 2 COVID-19 patients.** The clinical data, the lung, liver, renal, cardiac functions and homeostasis or blood vessels related data are shown according to the group to which they belong.

|  | **Group 1 (N=8)** | **Group 2 (N=16)** | **p-values** |
| --- | --- | --- | --- |
| **Age** | 54.8 [34;77] | 61.3 [35;85] | 0.23172 |
| **Gender (male)** | 75% (6/8) | 69% (11) | 1* |
| **Diabetes** | 12.5% (1/8) | 18.75% (3/8) | 1* |
| **Arterial hypertension** | 25% (2/8) | 62.5 (10) | 0.193* |
| **BMI** | 24.9 [19.7;28] | 29.2 [21.9;42] | **0.04482** |
| **ICU hospitalization** | 62.5% (5/8) | 100% (16) | **0.02767*** |
| **Death** | 12.5% (1/8) | 43.75% (7) | 0.1893* |
| **Lung function** | | | |
| **WHO Score ≤5** | 6 | 1 | **6.124e-05**** |
| **WHO Score >6** | 2 | 15 |  |
| **pCO_2_** | 32.7 [25;36] | 37.3 [28;58] | 0.17019 |
| **pO_2_** | 121.3 [61;254] | 106.5 [52;248] | 0.73994 |
| **SATO_2_** | 97 [94;100] | 97.8 [93;100] | 0.51573 |
| **Liver function** | | | |
| **HSI** | 29.6 [22.4;35] | 35.3 [24.7;48] | **0.04482** |
| **ASAT (UI/L)** | 50.8 [12;88] | 65.1 [31;150] | 0.35812 |
| **ALAT (UI/L)** | 27.4 [8;55] | 43.1 [11;148] | 0.19236 |
| **Total bilirubin (mmol/L)** | 10.6 [6;22] | 13.4 [5;36] | 0.87777 |
| **gGT (UI/L)** | 75 [16;244] | 138.4 [23;518] | 0.14332 |
| **Prothrombin Ratio (%)** | 91.4 [75;103] | 73.3 [13;99] | **0.02491** |
| **Renal function** | | | |
| **Serum creatine peak (µmol/l)** | 198.1 [62;921] | 315.8 [58;900] | 0.08733 |
| **Serum Creatinine at admission (µmol/L)** | 97.4 [46;250] | 141.2 [46;448] | 0.23223 |
| **ACR/PCR** | 249.9 [60;491.1] | 320.8 [25.9;774.4] | 0.662 |
| **Na^+^/K^+^** | 2 [0.4;3.4] | 0.4 [0.1;1.2] | **0.01401** |
| **Urine Na+ (mmol/l)** | 55.8 [15;90] | 17 [10;50] | **0.0075** |
| **Cardiac function** | | | |
| **Troponin (ng/mL)** | 11.3 [2.3;38.9] | 120.3 [3.6;453] | **0.00846** |
| **Hemostasis** | | | |
| **Fibrinogen (g/L)** | 7 [6.1;8.7] | 6.9 [5;9.2] | 0.69596 |
| **D-dimers (mg/L)** | 1309.2 [632;2707] | 2841.7 [738;8115] | 0.0983 |
| **Fibrin monomer (mg/mL)** | 6.6 [5;7] | 6.9 [5;7] | 0.4866 |
| **Blood vessels** | | | |
| **Angiopoietin1 (pg/mL)** | 22694.3 [2522.6;64929.6] | 17208.6 [2462.3;48800.7] | 0.65291 |
| **Angiopoietin2 (pg/mL)** | 5279.1 [1343.4;9550.9] | 8642.2 [1559.8;20782.2] | 0.17193 |
| **E-selectin (pg/mL)** | 39067.2 [13991.2;61126.4] | 57196.1 [24010;104214.1] | **0.03243** |
| **P-selectin (pg/mL)** | 60530.9 [22593;116179.6] | 79185.5 [29930.1;170939.5] | 0.13583 |
| **Ckit (pg/mL)** | 10741.9 [402.6;26085.4] | 3617.7 [402.6;9564.2] | 0.14069 |
| **VEGF (pg/mL)** | 279.6 [12.9;1073.3] | 189.6 [2.2;855.2] | 0.31963 |
| **FGFb (pg/mL)** | 34.8 [3.6;112.2] | 49.4 [11.2;179.7] | 0.4523 |
| **PlGF (pg/mL)** | 26.9 [11.4;37.1] | 35.9 [21.8;51.6] | **0.03823** |

All : Wilcoxon test. *: Fisher test; **: Cochran-Armitage trend test (more than 2 groups)

**Supplemental table 7: Quantification of molecules and cytokines in COVID-19 patients.** Values are shown in pg/mL (except CRP) for all COVID-19 patients or according to the group to which they belong.

|  | **Group 1 (N=8)** | **Group 2 (N=16)** | **p-values group 1 vs group 2** |
| --- | --- | --- | --- |
| **Immunology / Inflammation** | | | |
| **CRP (mg/L)** | 159.1 [69;248.9] | 204.3 [2.3;429] | 0.35028 |
| **HO1** | 4.4 [1.9;7.7] | 8.8 [4.7;14.5] | **0.00052** |
| **IL2Ra** | 74.3 [23.3;134.6] | 160.5 [52;417.1] | 0.07023 |
| **sCD163** | 96590.8 [39015.3;179781.4] | 146152.5 [12964.9;767709] | 0.9018 |
| **sIL6Ra** | 15750.7 [2637.3;28000.6] | 20294.9 [4832.2;49799.3] | 0.53584 |
| **sTNFR1** | 2112.4 [195.7;4360.1] | 5647.2 [498.4;32274.3] | 0.29912 |
| **sTNFR2** | 695.9 [178.8;1730.4] | 1778.6 [602.2;3941.3] | **0.00718** |
| **Cytokines** | | | |
| **IL1-α** | 0.8 [0;1.9] | 0.6 [0;1.9] | 0.89012 |
| **IL1-β** | 5.2 [0.5;23.6] | 5 [0.5;24.3] | 0.87825 |
| **IL1-ra** | 1621.2 [197.9;4346.4] | 3163.3 [469.3;18128.5] | 0.48958 |
| **IL-2** | 12.6 [1.6;38.5] | 18.3 [3.9;38.1] | 0.17772 |
| **IL-4** | 3.2 [1.5;4.5] | 5.5 [ 3;9.7] | **0,01307** |
| **IL-5** | 12.7 [0.9;43.9] | 19.3 [0.9;43.3] | 0.25591 |
| **IL-6** | 20.6 [1.9;42.4] | 105.9 [15.7;328.2] | **0.00026** |
| **IL-7** | 37.4 [11.5;55.1] | 60.4 [27.7;93.1] | **0.04298** |
| **IL-8** | 27.7 [1.5;85.4] | 40.6 [15.3;71.1] | **0.04325** |
| **IL-9** | 134.2 [78;199.8] | 158.5 [84.4;265.9] | 0.39106 |
| **IL-10** | 21.2 [0.1;120.2] | 16.4 [0.5;40.1] | 0.24452 |
| **IL-12** | 10 [2.1;21.9] | 11.6 [1.6;23] | 0.64503 |
| **IL-13** | 8.1 [1.2;20.5] | 7.3 [1.9;20.6] | 0.80633 |
| **IL-15** | 95.8 [7.8;320.2] | 104.3 [30.5;219.3] | 0.50896 |
| **IL-17** | 18.5 [4.4;41.9] | 31.9 [6.3;54.3] | 0.06584 |
| **IL-18** | 105.9 [21.7;319.8] | 194.1 [66.8;395.6] | 0.06835 |
| **IL-21** | 30.9 [0.1;156.6] | 0.1 [0.1;0.1] | 0.06829 |
| **IL-22** | 5.1 [0.1;19.1] | 6.3 [0.1;31.3] | 0.8778 |
| **IL-23** | 11.3 [0.1;78.8] | 0.1 [0.1;0.1] | 0.23006 |
| **IFN-γ** | 12.2 [1.4;30.2] | 59.3 [1.4;337.8] | 0.15754 |
| **CXCL10** | 5063.8 [467.1;12803.3] | 7431.2 [1930.8;19682.9] | 0.26356 |
| **CCL2** | 73.8 [5.2;192.5] | 194.4 [9.8;538.9] | 0.0926 |
| **CCL3** | 3.8 [1;7.8] | 5.9 [3.1;12.4] | 0.08087 |
| **CCL4** | 60.6 [33.9;87.5] | 64.3 [49.5;76.7] | 0.4439 |
| **CCL5** | 7008.5 [686.6;19739.9] | 3986.2 [1587.7;8371.5] | 0.92844 |
| **TNF-α** | 52.3 [38.4;78.3] | 87.2 [25.1;145.1] | **0.04994** |
| **Eotaxin** | 65.9 [16;139.5] | 94.8 [20.5;227.4] | 0.15312 |

All : Wilcoxon test
